# Optimizing Stratification in Binary Colloidal Supraparticles

**DOI:** 10.1002/advs.76284

**Published:** 2026-06-28

**Authors:** Frederic Rudlof, Silas Wolf, Sonja Schaller, Jonathan Martín González, Carsten Schilde, Nicolas Vogel

**Affiliations:** ^1^ Institute of Interfaces and Particle Technology Friedrich‐Alexander‐Universität Erlangen‐Nürnberg Erlangen Germany; ^2^ Institute for Particle Technology Technische Universität Braunschweig Braunschweig Germany

**Keywords:** CFD‐DEM simulation, diffusiophoresis, spray‐drying, structure formation, supraparticles

## Abstract

Stratified binary supraparticles enable functional architectures for applications such as photonics, pharmaceutics, and additive manufacturing. However, the extent of stratification under predefined particle size ratios and compositions remains unclear. Here, stratification in spray‐dried supraparticles from charge‐stabilised binary mixtures of colloidal primary particles under such boundary conditions is quantified via SEM image analysis and confocal microscopy. These experiments reveal the existence of optimal combinations of size ratios and volume fractions that produce maximal stratification, contrasting the intuitive picture that stratification increases with increasing size ratios. For low volume fractions of small particles, stratification is maximal with size ratios of ∼3–5 and shifts to lower size ratios with increasing relative volume fractions. CFD‐DEM simulations reproduce this trend and provide a contour plot of the extent of stratification as a function of particle size ratio and volume fraction. The simulations further suggest that stratification is driven by migration of small particles through interstitial channels between large particles at lower small particle concentrations, while colloidal diffusiophoresis becomes prominent at higher small particle contents. The understanding of stratification under experimental boundary conditions provides a framework for the predictive design of hierarchical supraparticle architectures with tailored internal structure.

## Graphical abstract

Supraparticles from binary particle populations show stratification when dried at high Péclet numbers. In spray‐dried systems at high initial particle concentration and fast drying conditions optimal combinations of particle size ratios and volume fractions that produce maximal stratification are found both in experiment and simulation, contrasting the intuitive picture that stratification increases with increasing size ratios.

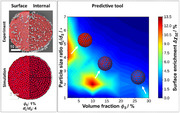

## 1 Introduction

Supraparticles are spherical, microscale particle aggregates [1]. They can be obtained from the confined assembly of colloidal primary particles within droplets [2]. Supraparticles combine the unique properties of nanoscale building blocks by colocalization of multiple components [3, 4, 5] or even provide additional properties arising from coupling effects [6, 7] or emergent functionalities [8, 9, 10, 11], such as tailored porosity for catalysis and gas sorption [8, 12, 13, 14], increased powder flowability [15, 16] or structural coloration [17, 18, 19, 20].

Several self‐assembly methods have been developed to fabricate such supraparticles, depending on the accuracy and throughput that is needed for a desired system. These include emulsion‐based routes, such as membrane emulsification [21], or droplet‐based microfluidics [17, 22, 23], and dry techniques like sessile droplet drying [24, 25, 26] or spray drying [3, 27, 28, 29, 30]. While droplet‐based microfluidics enables the preparation of highly uniform model systems under thermodynamic control [22, 23], spray drying provides large quantities of supraparticles in a scalable and fast process [30].

An interesting strategy to tailor the internal structure of supraparticles and thus further expand their functionality is the use of binary mixtures of primary particles with distinct sizes. Under slow drying conditions, such binary systems are used to study more complex crystal structures [31, 32]. Rapid drying conditions, for example, found in spray drying, enable the formation of hierarchical internal architectures such as core–shell‐like structures [15, 25, 29, 33, 34], which is particularly relevant for applications relying on internal transport, such as the control of release profiles of active pharmaceutical ingredients [35], or catalytic activity [36].

Such internal organization results from stratification effects that occur under non‐equilibrium conditions found with rapid drying conditions of colloidally stable dispersions, where the motion of the receding interface is faster than the diffusive motion of the individual particles. In this scenario, characterized by a Péclet number Pe ≫ 1, concentration gradients towards the interface of the droplet build up by accumulation of particles at the receding boundary [27, 37]. Particle motion within the drying dispersion droplet ultimately leads to stratification, which is caused by the interplay between hydrodynamic drag [38, 39, 40, 41, 42] (outwards directed) and osmotic pressure gradients (inwards directed), which is driven by the radial concentration gradients [37, 38, 43]. More recent studies indicate that the mechanism is governed by gradients in chemical potential [39, 43]. While this is equivalent to the osmotic pressure gradient approach for ideal single‐component descriptions [44], the chemical potential description also captures the cross‐interaction contributions that drive size‐asymmetric stratification in mixtures [38, 39, 40, 43]. At high Péclet numbers, the particle concentration gradients are pronounced, and large particles are transported more efficiently towards the droplet center due to their larger gradient in chemical potential, while smaller particles tend to remain closer to the interface [25, 27, 45, 46]. This phenomenon is referred to as *colloidal diffusiophoresis* and causes inhomogeneous particle distributions after full consolidation: the small particle population tends to enrich near the droplet surface, while the larger particle fraction is concentrated near the supraparticle center [3, 25, 47]—a phenomenon analogous to “small‐on‐top” arrangements observed in drying films [42, 43, 45, 48]. In addition, smaller particles can also migrate through the voids formed by a matrix of the pre‐arranged larger particles. This transport further increases stratification in binary systems with large size differences compared to pure diffusiophoretic effects [33].

Numerical modelling enables a detailed understanding and prediction of the formation dynamics of such structures. In the context of thin film drying, both continuum approaches—such as density functional theory (DFT) or analytical scaling models [38, 40, 43, 49, 50]—and particle‐based methods such as molecular dynamics models (MD) based on Brownian or Langevin dynamics [25, 39, 45, 51, 52] have already been employed to study stratification behavior. Similar modelling strategies have been adapted for drying colloidal droplets, including MD and discrete element method (DEM) used to simulate particle motion and segregation [53, 54, 55, 56, 57]. In particular, coupling of DEM with computational fluid dynamics (CFD) enables the simulation of structure formation in drying droplets by taking into account both fluid dynamics and particle motion [46, 55, 56, 57], with a high potential to capture stratification effects during spray drying of binary mixtures.

The existing body of literature suggests that particle segregation is maximized with increasing size ratio of the involved particle populations [3, 25, 46]. However, from a practical point of view, the design of a supraparticle structure is often limited by the availability of the different primary particles (i.e., that the dimensions of one or both particle populations are predetermined), and by a desired final composition of the supraparticle (i.e., to form particles with a predefined mixing ratio of both components). How segregation effects can be most efficiently invoked under such boundary conditions remains unknown.

Here, we address this knowledge gap and quantify segregation in binary mixtures of well‐defined primary particles under such boundary conditions in a combined experimental and numerical approach. First, we maintain a fixed relative volume fraction of both populations and systematically vary the size ratio. Second, we fix the size ratio and vary the relative volume fraction of the two particle types. After validating the numerical method with the experimental results, simulations are then further used to explore the driving factors leading to stratification. Ultimately, this strategy provides conditions to achieve optimal stratification for a desired volume‐ and size ratio and thus enables the predictive design of complex supraparticles.

## 2 Results

### 2.1 Surface Composition of Supraparticles with Varied Size and Volume Ratios

**FIGURE 1:**
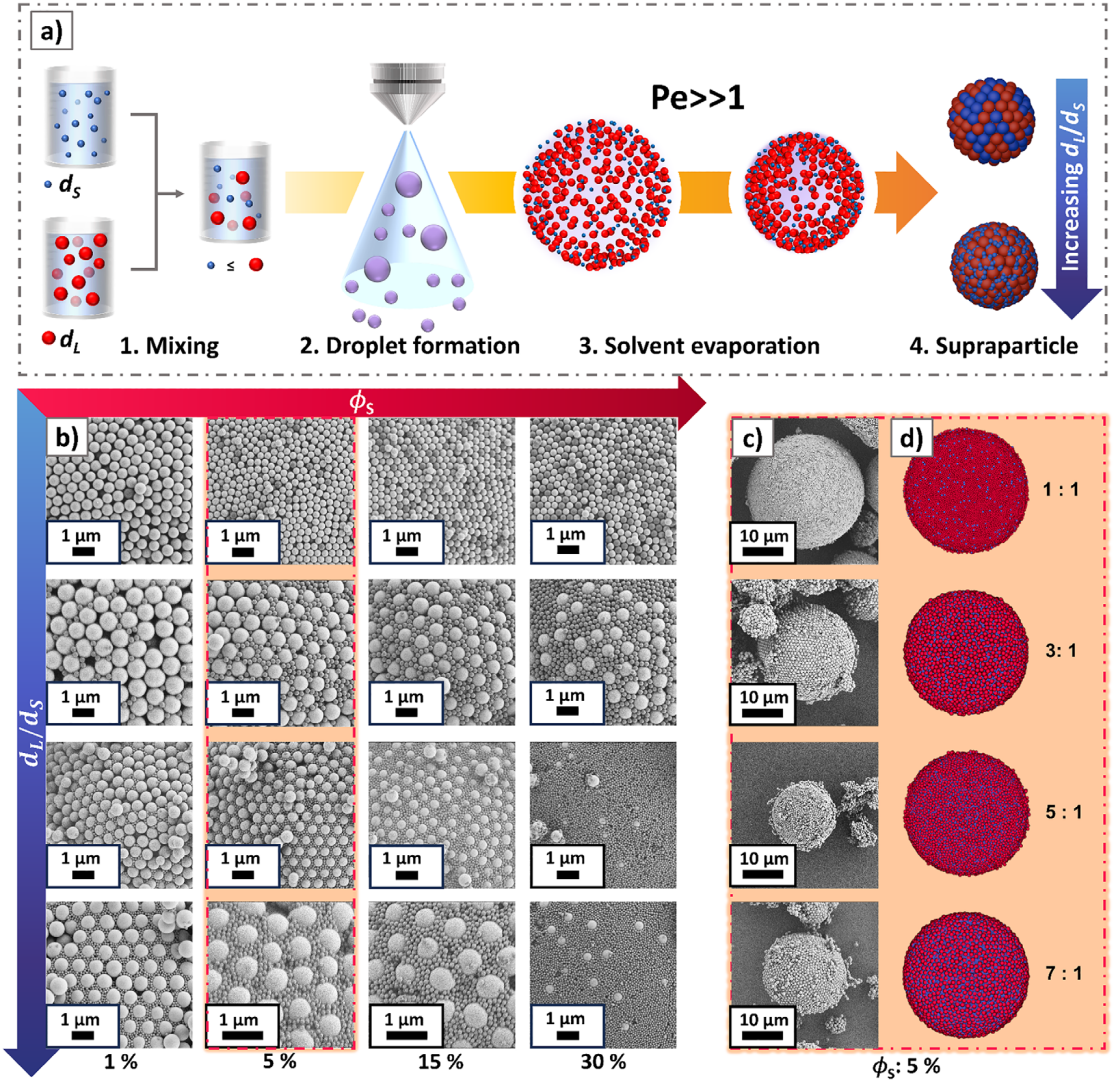
Fabrication of binary supraparticles with controlled size ratios and relative volume fractions. (a) Schematic illustration of the supraparticle formation process. (b) SEM images showing the surface structure of binary SiO_2_:PS supraparticles. (c,d) Comparison of experimental (c) and simulated (d) supraparticle morphologies at a constant $\phi_{S}=5\%$ and increasing size ratios *d_L_*/*d_S_ *= 1–7.

We start our study by fabricating supraparticles from mixtures of silica (SiO_2_, large particle population) and polystyrene (PS, small particle population) primary particles (see Figure S1) by spray drying (Figure 1a). Based on previous work, we obtained dense, fully consolidated supraparticles at solid concentrations of ∼25 – 30 vol.% [15, 58]. Therefore, we fix the solid content of the dispersion at 28 vol.% for all experiments. We systematically vary the diameter of the small population and the large particles to obtain a broad range of size ratios *d_L_*/*d_S_* of 1 – 7 in our binary systems (details in Table S1). In addition, we also vary the relative volume fraction of the small particles, $\phi_{S}=V_{P,S}\;/\left(V_{P,S}+V_{P,L}\right)$ with *V*_*P*,*S*_ being the total volume of small particles and *V*_*P*,*L*_ the total volume of large particles, from 0.01 to 0.3 (i.e., 1 to 30 vol.% of small particles). We estimate that the Péclet numbers for our spray drying systems are sufficiently large (Pe ≫ 1) for both particle populations (see Table S1), so that stratification, caused by (i) the solid accumulation at the surface region of the droplet driven by the fast movement of the fluid–fluid interface; and (ii) the different inward forces for both particle populations, can be expected.

To complement our experiments, we use numerical simulations of the drying process to gain access to particle‐scale dynamics and segregation mechanisms that are not directly observable experimentally. Our DEM‐based model considers contact and Derjaguin‐Landau‐Verwey‐Overbeek (DLVO) forces between individual particles, while the surrounding fluid and the dynamic fluid–fluid interface are captured on a continuous scale via the volume‐of‐fluid (VOF) method (for more information, see Section S1) [46, 55]. Particle–fluid and particle–interface interactions are represented by drag and capillary forces, implemented via unresolved CFD‐DEM coupling [46, 55].

Representative scanning electron microscopy (SEM) images of the resulting supraparticles are shown in Figure 1b (see Figure S2 for the full dataset). The formed supraparticles exhibit a spherical morphology (Figure 1c) with diameters around 17 µm (Table S2). From these SEM images, it is evident that both *d_L_*/*d_S_* and $\phi_{S}$ influence the surface composition of the supraparticles. Across all size ratios, an increase in $\phi_{S}$ or *d_L_*/*d_S_* results in enhanced surface coverage by small particles. The same trend is found in CFD‐DEM simulations, shown for $\phi_{S}=5\%$ in Figure 1d (full dataset shown in Figure S3). The results seemingly indicate the enrichment of small particles towards the droplet interface during drying caused by stratification. However, one also needs to consider that the fraction of small particles within the entire bulk of the supraparticle (χ_*S*,*b*_) increases both with their relative volume fraction and size ratio as:

(1) $$\chi_{S,b}=\frac{\phi_{S}\;{\left(\frac{d_{L}}{d_{S}}\;\right)}^{3}}{1-\phi_{S}+{\left(\frac{d_{L}}{d_{S}}\right)}^{3}\phi_{S}}\;$$

We use the subscript χ_*S*,*b*_ to indicate the bulk number fraction of small particles, since, as we will see, the number fraction at the surface, which we will denote χ_*S*,*s*_ in the following, will differ from this value because of stratification. According to Equation (1), changes in relative volume fraction cause a linear increase in number fraction while variations in size ratio result in a more pronounced increase due to the cubic relationship. For instance, when using $\phi_{S}=5\%$, a size ratio of *d_L_*/*d_S_* =  5 yields ∼7 small particles per one large particle while for $\phi_{S}=30\%$, this increases to > 50 (see Table S3). Thus, increasing $\phi_{S}$ results in a higher fraction of small particles within the supraparticle itself. In the same manner, for $\phi_{S}=5\%$, changing the size ratio from 3 to 7 leads to a rise in the number ratio from ∼1.4 to > 18. Therefore, the mere increase in the fraction of smaller particles at the surface observable in the SEM images does not necessarily result from an enrichment effect, that is, from stratification during the formation process, but may simply be caused by an increase in their overall number.

**FIGURE 2:**
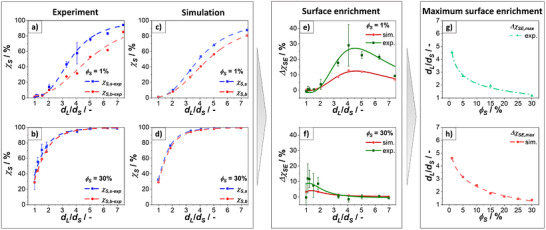
Determination of the surface enrichment of small particles, Δχ_*SE*_, in binary supraparticles with defined size ratios and relative volume fractions of the small particle population. (a,b) Experimentally determined surface number fraction of small particles, χ_*S*,*s* − *exp*_, and bulk number fraction, χ_*S*,*b* − *exp*_, for $\phi_{S}=1\%$ and $\phi_{S}=30\%$, respectively. (c,d) Corresponding simulation results for the same conditions. Dashed lines indicate empirical fits. (e,f) Surface enrichment Δχ_*SE*_ as a function of particle size ratio for $\phi_{S}=1\%$ and $\phi_{S}=30\%$, respectively, for experimental (green) and simulated data (red). (g,h) Particle size ratios *d_L_*/*d_S_* with the highest surface enrichment as a function of relative volume fraction, determined from the maxima in the experimental and the simulated data shown in (e,f), respectively.

To assess the extent of stratification, therefore, requires a direct comparison of the surface composition with the bulk composition within the entire supraparticle. We quantify the surface composition, and, in particular, the number fraction of small particles at the surface (χ_*S*,*s*_) via image analysis of the SEM data. To distinguish from the simulation data, we label this experimentally determined number fraction at the surface χ_*S*,*s* − *exp*_. We then determine the bulk composition in experiment, i.e., the number fraction of small particles in the entire supraparticle, from thermogravimetric analysis (TGA), where we can selectively combust the PS fraction within the supraparticle powders (see Section S2, Figure S4). This bulk number fraction is subsequently denoted χ_*S*,*b* − *exp*_. Figure 2a–d compares the evolution of these two number fractions as a function of size ratio in experiments and simulations, representatively for $\phi_{S}=1\%$ and $\phi_{S}=30\%$ (see Figure S5 for complete dataset). Both the experimental and the simulated data show a stronger increase of χ_*S*,*s*_ than of the corresponding bulk number fraction χ_*S*,*b*_ with increasing size ratio, suggesting enrichment of small particles at the interface. We fit both χ_*S*,*s*_ and χ_*S*,*b*_ based on a fitting function derived for the change in number composition with changing size ratios to interpolate data points (details in Section S2). These fits are shown as dashed lines in Figure 2a–d.

We now quantify the extent of stratification by computing the excess particles at the surface, which we term *surface enrichment* Δχ_*SE*_, via the number fractions on the surface and in the bulk:

(2) $$\Delta \chi_{SE}=\chi_{S,s}-\chi_{S,b}$$

Figure 2e,f shows the evolution of Δχ_*SE*_ as a function of particle size ratio for two representative relative volume fractions of $\phi_{S}=1\%$ and $\phi_{S}=30\%$ (complete datasets in Figure S5). In both cases, Δχ_*SE*_ initially increases with increasing size ratio, but eventually decreases at higher size ratios. Thus, surprisingly, we find a maximum in surface enrichment at intermediate particle size ratios. In addition, the extent of surface enrichment, is more pronounced for $\phi_{S}=1\%$ compared to $\phi_{S}=30\%$.

From the maximum in the curves (see Figure 2e,f), we extract the size ratio at which the surface enrichment is maximal. Figure 2g,h shows the evolution of the size ratios producing maximal enrichment as a function of the relative volume fraction of small particles. For $\phi_{S}=1\%$, the experimental maximum is found at *d_L_*/*d_S_* ≈ 4.59, in agreement with simulations that yield a value of 4.61, with a deviation of only ∼0.5% compared to the experiment. At $\phi_{S}=30\%$, both experiment and simulation predict lower size ratios of 1.22 and 1.36, respectively, with a deviation of ∼10%. Despite these small differences, the qualitative agreement suggests that the simulation model captures the essential features of the drying and segregation process. However, while simulations and experiments exhibit similar trends and peak positions, the experimentally observed Δχ_*SE*_ is consistently larger than that predicted by the model across all tested size ratios and relative volume fractions. The discrepancy becomes particularly evident at intermediate size ratios (e.g., $d_{L}/d_{S}\;∼3-5$), where experimental Δχ_*SE*_ values exceed simulation results by up to ∼15% at $\phi_{S}=1\%$ (see Figure 2e). This deviation suggests that the simulations underestimate interfacial accumulation.

Both the experimental (Figure 2g) and the simulated data (Figure 2h) suggest that with increasing relative volume fraction, the size ratio at which maximal surface enrichment, Δχ_*SE*,*max*_, is observed shifts to lower values. In other words, there are ideal size ratios to maximize surface enrichment, which depend on the total composition of the system.

### 2.2 Determination of Internal Stratification

Next, we investigate the spatial extent of the stratification within the supraparticle via the radial distribution of small particles. To this end, we use cross‐sectional SEM image analysis of fractured particles to quantify the number of small particles along the cross‐section. This particle‐resolved quantification enables a direct comparison with simulation data where the individual particle positions are readily available.

**FIGURE 3:**
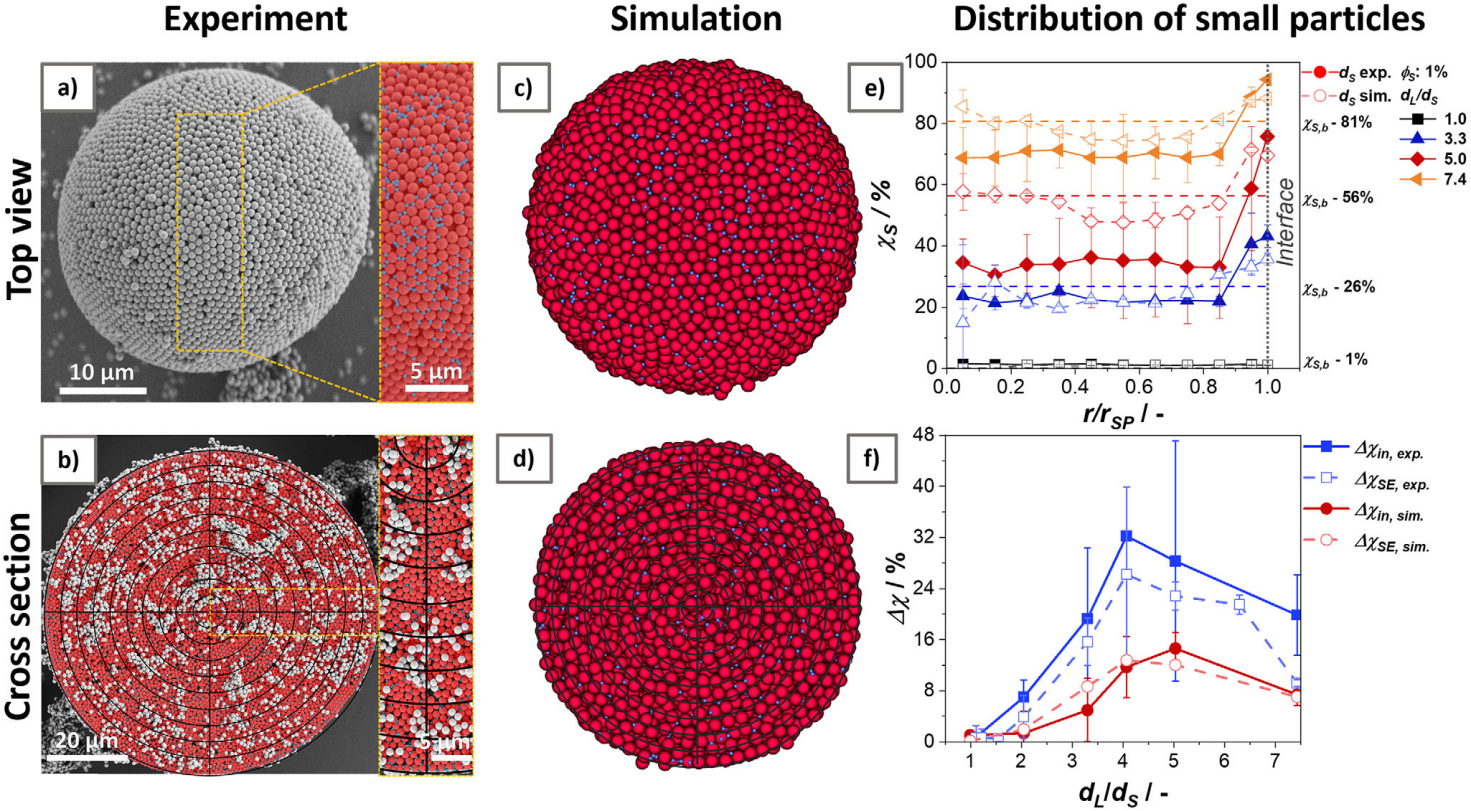
Evaluation of internal stratification of binary supraparticles based on image analysis. (a,b) Top‐view and cross‐sectional SEM images with a magnified area (b, right side) of a fractured supraparticle with $\phi_{S}=1\%$, *d_L_*/*d_S_* =  3.3, segmented into 11 concentric radial shells from the centre to the surface. The large particles are color‐coded in red, small particles in blue. (c) Top‐view of a simulated supraparticle with corresponding composition. (d) Cross‐section of the same simulated supraparticle, showing the spatial distribution of small (blue) and large (red) particles. (e) Radial distribution of the number fraction of small particles, χ_*S*_, as a function of normalised supraparticle radius *r*/*r_SP_*, obtained from experimental image analysis (solid symbols) and simulations (open symbols). Horizontal dashed lines indicate χ_*S*,*b*_. (f) Number contrast Δχ_*in*_ and surface enrichment Δχ_*SE*_ for experiments (blue) and simulations (red).

Figure 3a shows a top‐view SEM image of a representative supraparticle with $\phi_{S}=1\%$ and *d_L_*/*d_S_* =  3. Figure 3b shows a corresponding supraparticle cross‐section from the same sample, cleaved by mechanical fracture (see Figure S6 for additional data). To facilitate the identification of the particle populations, we color‐coded the small particles in blue and the large particles in red, similar to the color code used in simulations. Note that fracture‐induced debris as well as particles not belonging to the central plane of the cross‐section are visible in the image. For quantitative analysis, only particles within the plane of the cross‐section were considered. Particles were excluded if they (i) appeared isolated and sitting on top of this central plane, (ii) were located below the plane of the cross‐section (visible, e.g., through cavities in the plane), or (iii) exhibited irregular shapes indicative of fracture fragments that were redeposited on top of the cross‐sectional plane. For the small particles, only those visible in the top layer belonging to the main cross‐sectional plane were considered; underlying small particles could not be reliably determined and therefore excluded. Excluded particles are shown in grey in Figure 3b. The cross‐section was segmented into 11 concentric radial shells, ranging from *r*/*r_SP_* =  0.0 at the core to *r*/*r_SP_* =  1.0 at the surface. Figure 3c,d shows the top (c) and cross‐sectional views (d) of a simulated supraparticle formed under the same parameters. The same radial segmentation was applied in the simulation to allow direct comparison with experimental data.

We use image analysis to quantify the radial distribution of both particle populations in the experiments and average over three different cross‐sections to increase statistical significance. Figure 3e shows the radial distribution of the number fraction of small particles, χ_*S*_, for four size ratios between *d_L_*/*d_S_* =  1.0 and *d_L_*/*d_S_* =  7.4 for the experimental data (solid symbols) and the simulation data (open symbols). The expected number fraction of small particles based on the mixing ratio in the initial dispersion, χ_*S*,*b*_, is indicated by a dashed line in the color corresponding to the different samples. In all cases, we used a relative volume fraction of small particles of $\phi_{S}=1\%$ to keep the overall population of small particles comparatively small and thus enable accurate quantification of particle numbers.

The radial distribution of small particles in both simulations and experiments shows stratification for all samples with *d_L_*/*d_S_* > 1, evidenced by a depletion of small particles in the core and an enrichment near the surface compared to the expected number fraction. Quantitatively, the experimental data show an increase in χ_*S*_ for *r*/*r_SP_* > 0.8, while simulations indicate this onset already at *r*/*r_SP_* > 0.7. These findings are in agreement with previous studies reporting stratification at ∼70%−80% of the particle radius [25, 59].

With increasing size ratio, the depletion of the small particles in the core region becomes more pronounced. Interestingly, however, the maximal enrichment of small particles in the near‐surface region does not follow this trend but increases initially up to a size ratio $d_{L}/d_{S}∼5$ and subsequently levels off. This behavior reflects the results of the surface enrichment discussed above. Figure 3f quantitatively compares the degree of surface enrichment, Δχ_*SE*_, and internal stratification as a function of particle size ratio ($\phi_{S}=1\%$ for all cases). The degree of internal stratification is expressed by the number contrast of small particles Δχ_*in*_:

(3) $$\Delta \chi_{\mathrm{in}}=\chi_{S,0.95}-\chi_{S,0.15}$$

which represents the difference in small particle number fraction determined for the surface‐near region (χ_*S*,0.95_, determined at a radius of *r*/*r_SP_* =  0.95) and the small particle number fraction close to the core (χ_*S*,0.15_, determined at *r*/*r_SP_* =  0.15). In both experiments (blue data points) and simulations (red data points), an increase in stratification is evident by the increase of both surface enrichment, Δχ_*SE*_, and number contrast, Δχ_*in*_, up to $d_{L}/d_{S}∼4$, beyond which the values decrease again. Again, the agreement of internal and surface segregation points to an intermediate size ratio that provides conditions for the most efficient stratification.

Figure 3e provides a rationale for the hindered stratification at higher size ratios: as the bulk number fraction of small particles rapidly increases with increasing size ratio, it eventually becomes so high (see value of χ_*S*,*b*_ =  81% for *d_L_*/*d_S_* =  7.4) that a large contrast between surface and bulk populations cannot build up. In other words, to establish a large contrast between surface and core, it is necessary to maintain a comparatively low number fraction of small particles in the bulk.

Overall, the simulation results show qualitative agreement with the experimental trends in stratification, both in terms of surface‐based analysis and internal particle distribution. This indicates that the simulation model provides a robust representation of the dominant structure formation mechanisms resulting from radial capillary forces, particle collisions, viscous drag, and an interplay of attractive and repulsive forces [42, 60]. However, we note that the simulations systematically underestimate stratification (see Figure 3f). This could be due to additional microscale phenomena that cannot be resolved within the unresolved CFD‐DEM coupling approach we used as our simulation tool. During droplet shrinkage, phenomena such as fluid backflow, droplet deformation, rearrangement of particles due to lateral capillary forces and particle transport phenomena driven by thermal gradients (thermophoresis) were not captured [42, 60]. In particular, the exact transition point (“locking point”) at which a densified shell prevents further droplet shrinkage is difficult to reproduce. In addition, all processes occurring during this second drying stage, such as buckling, bubble formation, and capillary transport through emptying pores, were beyond the scope of the simulation tools employed in this study.

**FIGURE 4:**
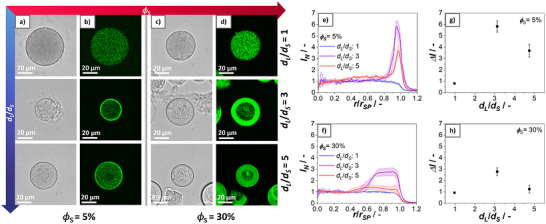
Distribution of the small particle population in binary supraparticles investigated by confocal microscopy with fluorescently labelled small particles. a–d) Confocal images of binary supraparticles with *d_L_*/*d_S_* =  1, 3, and 5 (top to bottom) in bright‐field (a,c) and fluorescence imaging modes (b,d) for $\phi_{S}=5\%$ and $\phi_{S}=30\%$. e,f) Normalized fluorescence intensity *I_N_* against the radial position within the supraparticle (*r*/*r_SP_*) for $\phi_{S}=5\%$ and $\phi_{S}=30\%$, respectively. g,h) Fluorescence contrast between surface and core, Δ*I*, as a function of particle size ratio *d_L_*/*d_S_*.

While image analysis of SEM cross‐sections allows quantification and comparison with simulation data, it is limited to small relative volume fractions of small particles ($\phi_{S}=1\%$) as otherwise the increasing number of small particles hinders accurate quantification. In addition, the radial extent of the accumulation region of small particles can only be coarsely assessed due to the binning into discrete steps. To analyze the degree and spatial extent of internal stratification at higher relative volume fractions of small particles, we resort to confocal microscopy with fluorescently labelled small particles to retrieve their distribution within the supraparticle. Figure 4a–d shows representative equatorial cross‐sections of three supraparticles formed with particle size ratios *d_L_*/*d_S_* =  1, 3, and 5 with two relative volume fractions of small particles ($\phi_{S}=5\%$ and 30%).

The optical bright‐field microscopy images show the presence of spherical, consolidated supraparticles in all cases (Figure 4a,c), which is crucial to avoid misinterpretation of data due to voids in the particle interior or buckled structures. The corresponding fluorescence microscopy images, shown in Figure 4b,d, reveal differences in the distribution of the small particles via their fluorescence signal. For a size ratio close to unity (*d_L_*/*d_S_* =  1), a homogeneous distribution can be observed. For the intermediate case of *d_L_*/*d_S_* =  3, the enrichment in near‐surface regions becomes evident. This enrichment is less pronounced for the largest size ratio *d_L_*/*d_S_* =  5, corroborating the evaluation of internal and surface enrichment discussed above. We analyze the distribution of small particles by radially averaging and plotting the fluorescence intensity as a function of its position with respect to the supraparticle radius (*r*/*r_SP_*) in Figure 4e,f. From these data, we evaluate the degree of stratification via the fluorescence contrast, Δ*I*, computed via the difference in fluorescence at the surface and in the core (Figure 4g,h).

This evaluation confirms the homogeneous distribution of the fluorescent particles, and thus Δ*I* =  1 for *d_L_*/*d_S_* =  1, corroborating that no stratification occurs when both species are of equal size [25, 42, 43, 45, 48]. For *d_L_*/*d_S_* =  3 and $\phi_{S}=5\%$, a sharp increase in fluorescence signal starting from *r*/*r_SP_* =  0.8 is evident. The contrast in fluorescence reaches Δ*I* =  5.8, indicating pronounced enrichment of small particles and thus effective stratification. At $\phi_{S}=30\%$, an intensity maximum persists but is less pronounced at the same size ratio (Figure 4f, purple). A broader enrichment with a plateau at $r/r_{\mathrm{SP}}∼0.6-1.0$ with a Δ*I* ≈ 2.8 is observed, indicating a reduced stratification efficiency.

For the larger size ratio *d_L_*/*d_S_* =  5, a lower increase in fluorescence intensity towards the near‐surface regions of the supraparticle is observed, in agreement with the evaluation of surface enrichment, Δχ_*SE*_, in Figure 2. For $\phi_{S}=5\%$, Δ*I* is reduced to 3.7, indicating that the stratification is less pronounced compared to *d_L_*/*d_S_* =  3. At $\phi_{S}=30\%$, Δ*I* only reaches 1.2, and the distribution of the smaller particles is smeared widely into the supraparticle interior.

In summary, the evaluation by confocal microscopy qualitatively indicates that the degree of stratification depends on both the size ratio and the relative volume fraction of small particles. In corroboration with the quantitative assessment of surface enrichment (Figure 2) and internal particle distribution (Figure 3), maximum enrichment is not observed for the largest size ratio but rather for intermediate values of *d_L_*/*d_S_* =  3.

### 2.3 Mechanism of Stratification

**FIGURE 5:**
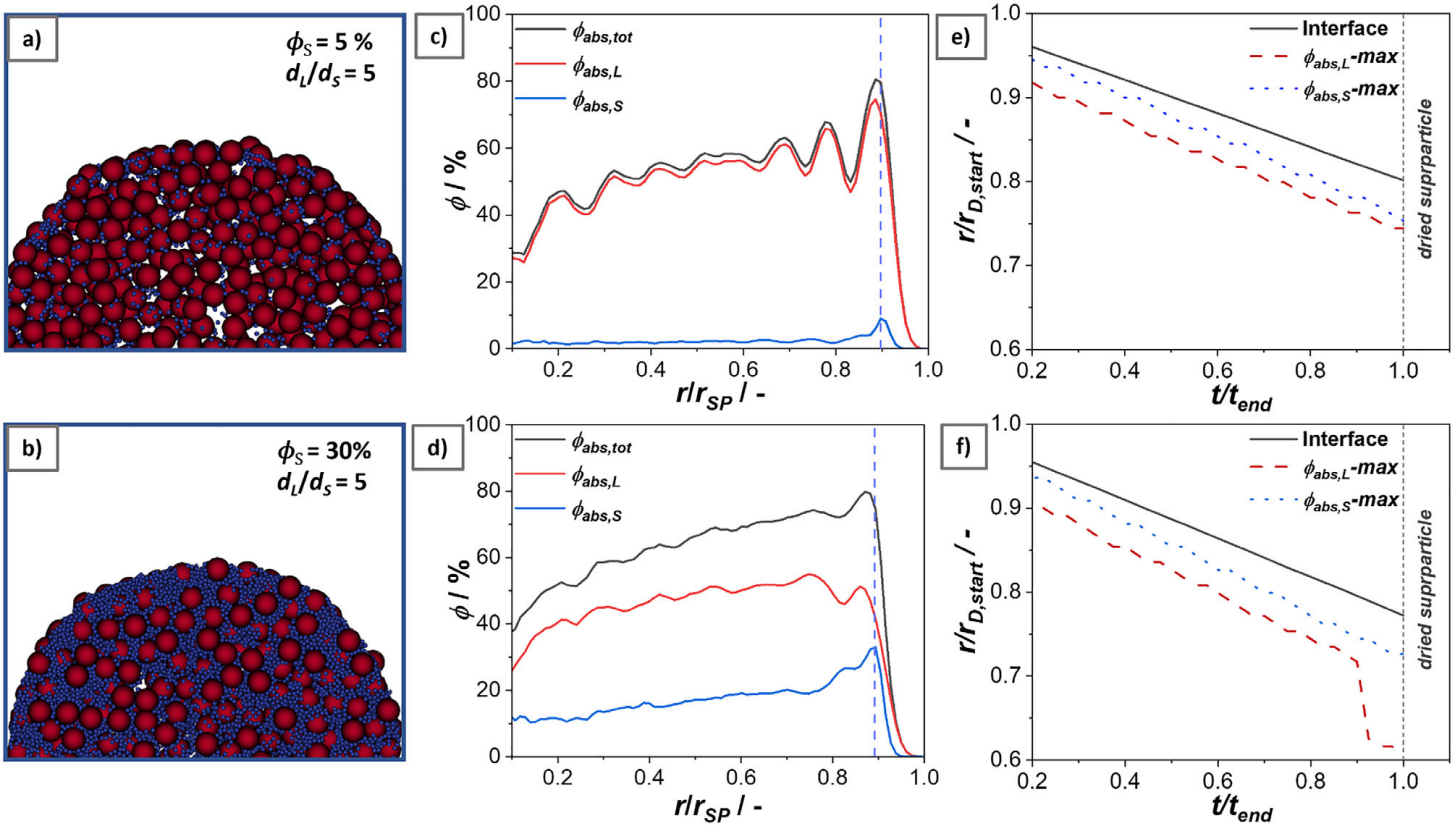
Distribution of both particle populations in stratified supraparticles formed under different initial relative volume fractions of small particles. Top row: $\phi_{S}=5\%$; bottom row: $\phi_{S}=30\%$. Both sets of simulations were performed with the same particle size ratio *d_L_*/*d_S_* =  5. a,b) Thin cross‐sectional slices of consolidated binary supraparticles from simulations, showing the radial distribution of large particles in red and small particles in blue. c,d) Radially resolved absolute volume fractions of small particles ($\phi_{abs,S})$, large particles ($\phi_{abs,L}$) and both particles ($\phi_{abs,tot}$). Dashed blue lines mark maximum enrichment of small particles. e,f) Evolution of maximum enrichment $\phi_{abs,L}-max$ and $\phi_{abs,S}-max$ in the course of the drying process for $\phi_{S}=5\%$ (e) and $\phi_{S}=30\%$ (f) along their radial position in respect to the initial droplet radius *r*_*D*,*start*_. *t/t_end_* = 1.0 shows the final, dried supraparticle.

To assess the mechanism behind the observed stratification, we analyze the particle distribution in simulations. Figure 5 shows snapshots at the end of the simulation runs, presented as thin cross‐sectional slice along with a thickness of 2 *d_L_* for supraparticles with a size ratio of *d_L_*/*d_S_* =  5 and two relative volume fractions, $\phi_{S}=5\%$ (Figure 5a) and 30% (Figure 5b). Figure 5c,d shows the radial distributions of the absolute volume fraction of the small ($\phi_{abs,S}$) and large ($\phi_{abs,L}$) particles, as well as the total solid volume fraction ($\phi_{abs,tot}$) for both simulations, respectively.

In both cases, an increase in the total solid volume fraction towards the supraparticle surface is observed (Figure 5c,d, black line). This implies that the formed supraparticle is less well packed within the center compared to the near‐surface regions. Such distributions are often observed in simulations of spray‐dried suspension droplets [60] and relate both to the drying process itself, which accumulates particles at the receding droplet interface [46, 61], and to the frustrated crystallization in the interior of the droplets [17]. In contrast, the outer regions allow for a better packing of particles in concentric layers [17, 32], which is evidenced by the radial fluctuations in $\phi_{abs,L}$.

Similarly, both cases show a distinct accumulation of small particles towards the surface, corroborating the experimental case in Figure 4 (Figure 5c,d, blue line). This increase occurs over a broader radial distance and is more continuous for the case of $\phi_{S}=30\%$ (Figure 5d), again in agreement with the smeared‐out fluorescence signal of the experimental counterpart in Figure 4.

The distribution of large particles, however, differs for the two types of supraparticles (Figure 5c,d, red lines). For $\phi_{S}=5\%$, the peak in the radial distribution of small particles coincides with the maximum of $\phi_{abs,L}$ (Figure 5c), implying that both large and small particles are present in high concentrations near the surface of the supraparticle. In contrast, for $\phi_{S}=30\%$, $\phi_{abs,L}$ decreases at the radial region in which the small particle population is maximal (Figure 5d). This behavior implies that the large particles are depleted from the surface. We analyze the dynamics of this depletion process by tracking the trajectories of the maxima of $\phi_{abs,L}$ and $\phi_{abs,S}$ in the course of the drying process (Video S1). Figure 5e,f shows the positions of both maxima (red dashed line and blue dotted line, for $\phi_{abs,L}$ and $\phi_{abs,S}$, respectively) relative to the position of the surface (black solid line) as a function of drying time. For $\phi_{S}=5\%$, both maxima remain close to the receding interface, while their relative positions with respect to each other are nearly constant, implying that both particles accumulate at the surface at early stages and subsequently move along with the receding surface (Figure 5e). In contrast, for $\phi_{S}=30\%$, the maximum of $\phi_{abs,L}$ becomes depleted from the interface during the final drying stage *t/t_end_* at 0.9, whereas the maximum of $\phi_{abs,S}$ remains located close to the interface (Figure 5f).

These differences imply different dominant mechanisms driving stratification. For $\phi_{S}=5\%$, the large particles maintain their positions at the interface, enriched by additional small particles that are present in interstitial voids (Figure 5c,e). This behavior suggests that channel‐driven segregation [62], where small particles migrate towards the surface, is the dominant mechanism in this case. For $\phi_{S}=30\%$, the depletion of large particles from the interface (Figure 5d,f) pinpoints to diffusiophoresis, that is, the preferential migration of large particles towards the interior of the supraparticle as the dominant mechanism. These differences suggest that the overall number of small particles determines the dominant mechanism. With smaller amounts of small particles, segregation occurs via enrichment of the small particle population at the surface, while with larger amounts of small particles, diffusiophoresis actively drives large particles away from the surface.

### 2.4 Predictive Design of Stratified Supraparticles

**FIGURE 6:**
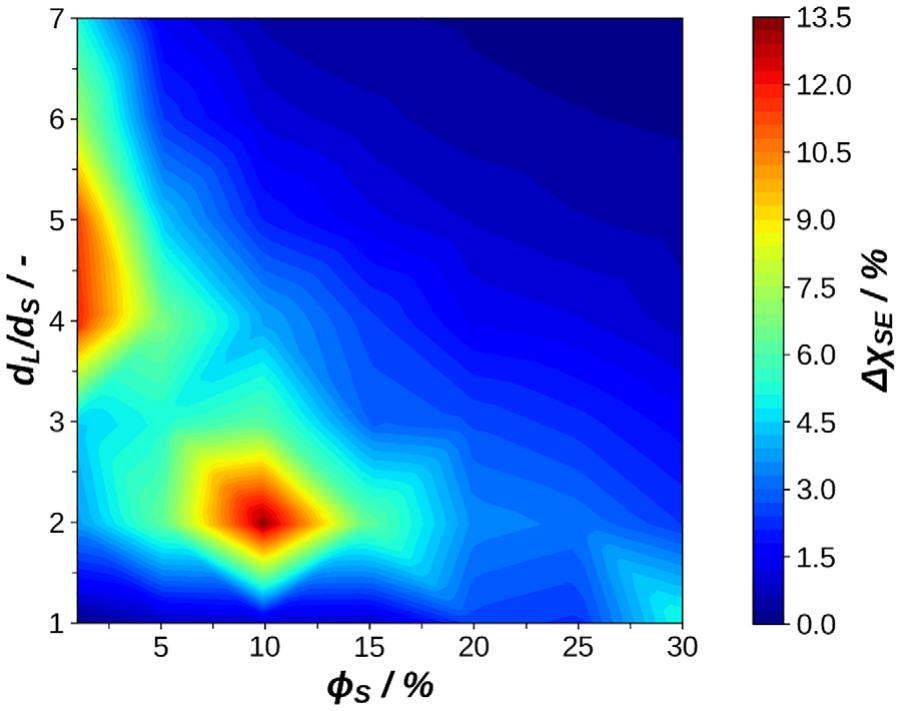
Contour plot showing the evolution of surface enrichment of small particles, Δχ_*SE*_, as a metric to quantify stratification in binary supraparticles, as a function of the relative volume fraction of small particles, $\phi_{S}$, and the size ratio, *d_L_*/*d_S_*, based on simulation results. The plot is available as an interactive tool via the Supporting Information.

As a summary of the parameter space explored in this study, we compile all simulations for the different particle systems and determine the surface enrichment of small particles, Δχ_*SE*_, as a measure for stratification efficiency in this system. Figure 6 shows these results in the form of a contour plot displaying Δχ_*SE*_ as a function of both the size ratio *d_L_*/*d_S_* and the relative volume fraction of small particles $\phi_{S}$. This contour plot contains data from 49 simulation runs, with linear interpolation applied between the values to generate a two‐dimensional map with 2500 data points. As for the simulations in the previous section, a constant diameter of the small particle population (*d_S_* = 270 nm) and a total initial volume fraction of both particles of 28% was used.

The contour plot in Figure 6 shows the evolution of Δχ_*SE*,*max*_ through the entire parameter space. With increasing $\phi_{S}$, the region with maximal surface enrichment shifts from larger to smaller *d_L_*/*d_S_*. A second notable feature of the contour plot is that the Δχ_*SE*,*max*_ seems to show two distinct maxima at $\phi_{S}\approx 1\%$ and $\phi_{S}\approx 10\%$. The observed maxima in the simulation show a difference in Δχ_*SE*,*max*_ of ∼50% compared with the minimum at $\phi_{S}\approx 5\%$. This pronounced difference in stratification by a change in composition underlines the importance of an accurate design of the particle system to tailor the degree of stratification. Both the evolution of Δχ_*SE*_ with varying *d_L_*/*d_S_* and $\phi_{S}$, as well as the presence of the two distinct regions with Δχ_*SE*,*max*_ are essentially similar in the experimental counterpart of the contour plot (Figure S7), albeit the minimum separating the two regions with Δχ_*SE*,*max*_ is shifted to a $\phi_{S}\approx 10\%$ in the experimental case.

The data compiled in the contour plot support the conclusions from the previous analyses: there are distinct maxima where Δχ_*SE*_ is largest, which depends on both the size ratio *d_L_*/*d_S_*, and the relative volume fraction of small particles $\phi_{S}$.

In other words, under experimentally realistic scenarios, where either the size ratio or the desired composition is fixed, the other parameter determines the degree of stratification that can be achieved. The combinations of both parameters that yield maximal stratification under the chosen conditions can be determined from the contour plot. We provide this contour plot as an interactive tool for the predictive design of binary supraparticles with tailored stratification, accessible via the Supporting Information (Section S3). This tool enables the prediction of the degree of surface enrichment for any binary system with a predetermined $\phi_{S}$, or for a desired relative volume fraction using a predetermined *d_L_*/*d_S_*.

## 3 Discussion

We now rationalize the observed stratification, the underlying mechanisms and their dependence on size ratio, *d_L_*/*d_S_*, and the relative volume fraction of small particles, $\phi_{S}$.

The key findings, summarized in the contour plot of Figure 6, are the following. First, the efficiency of stratification increases with increasing size ratio, but subsequently decreases. This behavior is robustly observed in experiments analyzing the surface of supraparticles (Figure 2), the distribution of particles by image analysis (Figure 3) and fluorescence microscopy (Figure 4), and in simulations (Figures 2 and 3). A maximum in stratification at intermediate size ratios is counterintuitive, as one would assume that the diffusiophoretic force driving larger particles inwards should increase with size ratio [38, 39, 43, 45, 48]. Second, stratification is coupled to the composition of the system: the maximum in stratification efficiency changes as a function of the relative volume fraction of the small particles. With increasing relative volume fraction, the size ratio for which maximal stratification is observed shifts to lower values. Third, the contour plot shows two broad regions with increased stratification efficiency: one at comparatively low $\phi_{S}$, and a second region at comparatively high $\phi_{S}$. These two maxima are separated by a region of $\phi_{S}$ where segregation is seemingly less efficient. This effect is seen both in simulation (Figure 6) and experiment (Figure S7).

We first discuss the maximum in stratification efficiency. Increasing the size ratio of particles increases the chemical potential difference driving diffusiophoretic stratification [40, 45, 48]. Furthermore, particles can seep through channels between the larger particles more easily. However, in our experiments with a fixed relative volume fraction of small particles, the number fraction of small particles rapidly increases due to the difference in particle volume (see above), so that the small particles eventually crowd the droplet interface [37, 63]. With increasing crowding, efficient stratification is prevented [49]. In other words, the number of small particles becomes so large that the movement of the large particles is hindered.

In addition, as seen in Figure 3, the large number of small particles prevents efficient stratification, as it decreases the maximum achievable number contrast between surface and bulk. Put simply, with too many particles present in the bulk, the concentration gradient is diminished, resulting in reduced surface enrichment.

Together, these two arguments also rationalize the observed shift in maximum stratification efficiency towards lower size ratios when the relative volume fraction of small particles is increased. The critical number fraction at which crowding occurs depends on the volume of initially available small particles: with increasing relative volume fraction, this effect occurs at decreasing size ratios. At large relative volume fractions ($\phi_{S}=30\%$), it dominates already at moderate size ratios and generally suppresses stratification. As a result, the system freezes in an intermediate state, consistent with the broad, smeared‐out and less intense region of small particle accumulation observed in the confocal microscopy images of Figure 4. To acquire maximum stratification, a balance between sufficient concentration and force gradients and interfacial crowding has to be found.

Notably, analysis of the data in Figure 6 suggests that across the different initial compositions (i.e., with varying $\phi_{S}$), the ideal size ratio providing maximal stratification is found when the ratio of small to large particles approaches unity, that is, $\chi_{S}/\chi_{L}∼1$. For example, simulations yield Δχ_*SE*,*max*_ when χ_*S*_/χ_*L*_ ≈ 0.99 at $\phi_{S}=1\%$ (corresponding to *d_L_*/*d_S_* =  4.6) and χ_*S*_/χ_*L*_ ≈ 1.08 at $\phi_{S}=30\%$ (corresponding to *d_L_*/*d_S_* =  1.4; see Figure S8). Similarly, in the experiments, Δχ_*SE*,*max*_ was found to be at χ_*S*_/χ_*L*_ ≈ 0.98 for $\phi_{S}=1\%$ and χ_*S*_/χ_*L*_ ≈ 0.78 for $\phi_{S}=30\%$. Although these values deviate from unity by ∼2% and ∼22%, respectively, the maxima are still surprisingly close to 1, suggesting that the populations are comparable in number, given experimental uncertainties in determining accurate particle numbers.

We tentatively rationalize this observation by a competition between mechanisms driving segregation, in particular channel‐driven stratification and diffusiophoresis, and counteracting effects such as jamming, crowding, and a saturation of the supraparticle with small particles. While the former are more effective with increasing numbers of small particles, the latter counteract the build‐up of stratification, leading to a balance, which is seemingly found for similar numbers of small and large particles. Yet, a quantitative theory underpinning this observation remains to be developed.

The association of Δχ_*SE*,*max*_, i.e., the conditions for optimal stratification, with similar particle numbers also underpins key features observed in the evolution of Δχ_*SE*_ as a function of size ratio, shown in Figure 2e,f. The peak of Δχ_*SE*_ is broad at low $\phi_{S}$ and becomes narrower with increasing $\phi_{S}$, consistently in experiments and simulations. This trend is based on the cubic dependence of χ_*S*_ on the size ratio combined with the influence of the volume ratio shown in Equation (1). Increasing $\phi_{S}$ shifts the size ratio required for Δχ_*SE*,*max*_ towards unity and simultaneously reduces the range of size ratios that yield comparable number fractions. As a result, both the peak position moves to smaller size ratios and the peak width narrows.

Finally, we hypothesize that the presence of the two distinct maxima in Δχ_*SE*_ observed in Figure 6 may relate to a change in the dominant mechanism underpinning the stratification. As discussed in the context of Figure 5, for lower numbers of small particles, channel‐driven stratification dominates (i.e. small particles migrate towards the interface), while at higher numbers of small particles, diffusiophoresis becomes more efficient (i.e., large particles migrate away from the interface). The minimum Δχ_*SE*_ separating the two regions of high Δχ_*SE*_ seemingly coincides with the transition between these two dominant mechanisms: While the overall maximum in surface enrichment remains at $\chi_{S}/\chi_{L}∼1$, the absolute value of the maximum is influenced by the different magnitudes of channel‐driven segregation at low $\phi_{S}$ and diffusiophoresis with increasing $\phi_{S}$.

A comparison with existing reports in the literature shows that our systems exhibit weaker segregation than reported for fast‐drying thin films [45] or sessile droplets [25]. We can attribute these differences to the time scales of the experiment. In both thin film and sessile droplet drying, despite having Pe ≫ 1, the overall time scales are longer compared to the supraparticles we prepare via spray drying, and the Péclet numbers are not identical. For example, for evaporating sessile drops, Liu et al. showed drying times τ_s_ ∼28.5 s and Pe = 180 of the small particles [25]. In contrast, our spray‐drying process exhibits τ_s_ ∼2.5 ms, a time difference of $∼10^{4}$, and Péclet numbers for the small particles Pe > 421. These differences imply that particle gradients are expected to be more pronounced in the experiments presented in this work, leading to an increased driving force for stratification. However, the sessile droplet system has more time to build up diffusiophoretic processes that displace the particles and, consequently, exhibits more pronounced stratification [25]. A similar conclusion was drawn by Tatsumi et al., who identified an optimum in the dependence of stratification strength on Pe [52]. Furthermore, we maintained a high initial volume fraction of the dispersion (28 vol.%) to ensure fully consolidated droplets and avoid buckling and hollow particles, which can form when spray drying at lower volume fractions. In this case, crowding and early‐onset jamming limit particle rearrangement and compress the time window in which diffusiophoretic stratification can develop even more [38, 39, 46, 49]. Together, these differences suggest that the Péclet number alone is not the sole descriptor for efficient stratification, but contributes along with the initial particle concentration and thus overall drying time.

Beyond Φ_S_ and *d_L_*/*d_S_*, stratification may be determined by the overall system size, and shifts in maximum stratification may depend on the size of the supraparticle and the overall number of constituent particles as well.

## 4 Conclusion

In this work, we investigated structure formation processes in binary colloidal supraparticles formed by the fast‐drying kinetics present in a spray dryer. Under such conditions, stratified non‐equilibrium structures are formed that exhibit an enrichment of the small particle population towards the surface of the supraparticle. We focused on experimentally realistic scenarios, in which either the size ratio of the particles in the binary mixture, or the composition of the system is predefined a priori.

Our results robustly show that under such conditions, distinct maxima in stratification are found for specific size ratios of the constituent particles, contrary to the expectation that stratification generally increases with increasing size ratio. Furthermore, the maximum in stratification depends on the relative volume fraction of the small particles, with a general trend that increasing the relative volume fraction causes a shift of the optimal size ratio for stratification to smaller values.

These trends are rationalized by the number of small particles present in the system, which must be sufficient to build up chemical potential gradients, and the occurrence of crowding with increasing numbers of small particles. Notably, our results indicate that optimal stratification conditions broadly coincide with the presence of equal numbers of both particle populations.

We compile all our data into a two‐dimensional design map that links the desired relative volume fraction and size ratio to the expected degree of stratification. This interactive tool provides a framework for the predictive design of supraparticle architectures with tailored stratification.

## 5 Experimental Section

### 5.1 Materials

Absolute ethanol (≥99.8%, NORMAPUR, VWR), technical ethanol (96%, VWR), ammonium hydroxide (NH_3_, 25%, NORMAPUR, VWR), tetraethyl orthosilicate (TEOS, ≥99%, GPR RECTAPUR, VWR), acrylic acid (99%, Sigma‐Aldrich), ammonium persulfate (≥98.0%, Sigma‐Aldrich), fluorescein isothiocyanate (FITC, isomer I, Sigma‐Aldrich), (3‐aminopropyl)triethoxysilane (APTES, ≥98%, Sigma‐Aldrich), sodium hydroxide (NaOH, ≥98%, Ph. Eur., USP, BP, pellets), aluminum oxide (90, neutral) were all used as received. Styrene (ReagentPlus, 99.9%, Sigma‐Aldrich) was washed with 10 wt.% aqueous NaOH solution and passed through an aluminum oxide column to remove the inhibitor 4‐tert‐butylcatechol. It was then stored no longer than 2 months at 8°C before use. For water, a Purelab Flex 2 (Elga Veolia) purification unit was used (18.2 MΩ·cm).

### 5.2 Synthesis of Silica Primary Particles

Colloidal silica particles were synthesized via the Stoeber method according to the literature [12, 64]. Water, absolute ethanol, and NH_3_ were mixed in a one‐liter, one‐necked round‐bottom flask and continuously stirred at 500 rpm. Separately, TEOS was mixed with absolute ethanol in a weight ratio of 1:1 and added. After 1 h, the solution turned pale white. The solution was stirred overnight at room temperature. The obtained particles were separated from the synthesis solution via centrifugation, followed by washing with water for 5 times. Afterwards, they were redispersed in water at a solid concentration of 30 vol.%. A total volume of 0.6 L was set for the synthesis. The concentration of the ethanolic synthesis solution was: 3.84 M water, 0.05–2 M NH_3_, and 0.14 M TEOS. Particle size was adjusted by changing the ammonia concentration: 0.05 M for 130 nm, 0.12 M for 270 nm, 0.20 M for 390 nm, 0.32 M for 500 nm, 1.44 M for 750 nm, 1.71 M for 900 nm, and 2 M for 1200 nm (see Figure S1).

### 5.3 Synthesis of Fluorescent Silica Primary Particles

Fluorescent SiO_2_ particles were synthesized via a modified Stoeber method reported in literature [65]. First, a 20 mL glass vial was light‐sealed with aluminum foil. FITC (6.38 mg, 1.64 × 10^−5^ M) was added to the vial. Absolute ethanol (10 mL) was then added, and the mixture was placed in an ultrasonic bath for 5 min. The vial was sealed with parafilm and flushed with argon. Subsequently, APTES (17.14 µL, 7.32 × 10^−5^ M) was added in excess, and the solution was stirred overnight at 500 rpm.

8.63 mL of the resulting APTES–dye conjugate solution in ethanol was added to 0.14 M of TEOS to achieve a doping concentration of 0.1 wt.% APTES‐dye conjugate in the final silica matrix. Additional ethanol was added to the TEOS/APTES‐dye mixture to reach a 1:1 weight ratio of TEOS to ethanol. The TEOS/APTES‐dye/ethanol solution was then introduced into a mixture of ethanol, water, and NH_3_ according to the silica synthesis protocol. The combined reaction mixture was stirred overnight. The resulting fluorescent silica particles were purified by centrifugation with 5 consecutive washing steps with water. The received fluorescent silica particles showed comparable particle sizes to pure SiO_2_ colloids.

### 5.4 Synthesis of Polystyrene Primary Particles

Monodisperse polystyrene particles were synthesized via a modified surfactant‐free emulsion polymerization, as reported previously [66]. In short, water (980 mL) was added to a two‐liter, three‐necked round‐bottom flask and heated to a nominal temperature of 90 °C via a heat block reaching an internal temperature of 79 °C. During heating, the mixture was constantly flushed with nitrogen while using a reflux condenser to prevent evaporation and stirring at 500 rpm. Reaching stable internal temperature after 2 h, styrene was added. At 10 min intervals, 0.4 g of acrylic acid and 0.4 g of ammonium persulfate, each dissolved in 5 mL of water, were added. After 24 h, the nitrogen flow and heating were stopped, and the colloidal dispersion was left to cool under stirring. The dispersion was filtered through a KimWipe (Kimberly‐Clark). The particles were then cleaned via five centrifugation‐redispersion cycles using water as dispersion medium. Then, they were dispersed in water at a solid concentration of 30 wt.%. The particle size was adjusted between 130 and 650 nm by varying the styrene amount from 25–50 g (see Figure S1).

### 5.5 Fabrication of Supraparticles

For the preparation of defined binary supraparticles, dispersions of SiO_2_ and PS or fluorescent SiO_2_ colloids were each concentrated to a total solids content of 28 vol.% before spray drying using particle densities of ρ_*PS*_ = 1.05 g mL^−1^ [67] and ρ_*SiO*2_ =  2.0 g mL^−1^ [65, 68, 69]. The particle dispersions were subsequently mixed in a defined relative volume fraction $\phi_{S}=V_{P,S}\;/\left(V_{P,S}+V_{P,L}\right)$, with *V*_*P*,*S*_ being the volume of small particles and *V*_*P*,*L*_ the volume of the large particles. $\phi_{S}$ of 1%, 5%, 15%, and 30% were selected, and the binary mixtures were ultrasonicated for 5 min, followed by mixing via a vortex shaker (VORTEX Genius 3, IKA) for 2 min to ensure a homogeneous mixture. All supraparticle samples were then produced with a spray dryer (B290 Mini, BÜCHI Labortechnik) under nitrogen atmosphere. All colloidal mixtures were prepared immediately before spray drying. The colloidal dispersion used as feed was atomized using a co‐current flow two‐fluid nozzle (diameter of 1.4 mm), at a gas flow of 357 L h^−1^ and feed flow of 3.4 mL min^−1^. The aspirator flow was kept at 35 m^3^ h^−1,^ and the inlet temperature was set to 120 °C. The as‐prepared supraparticles were then collected.

### 5.6 Characterization of Particle Size and Surface Morphology

The particle size distributions of primary particles were measured via SEM size distribution (Gemini 500, Zeiss) with an InLens detector, at an acceleration voltage of 0.5 kV, an aperture size of 10 µm, and a working distance of 2.9 mm. At least 200 primary particles were analyzed. For supraparticles, an SE2 detector with an acceleration voltage of 1.0 kV, an aperture size of 10 µm, and a working distance of 6.7 mm was used. Surface number fractions of supraparticles were determined by manual particle counting from top‐view SEM images using ImageJ. For each composition, at least three different supraparticles were evaluated. To this end, an even surface region of each supraparticle was selected at a magnification that allowed a reliable distinction between *d_S_* and *d_L_*. Depending on the composition and the size ratio, the total counted number of primary particles per supraparticle surface area ranged between 100 and 4000. Only particles belonging to the visible outermost continuous surface layer were considered; particles visible through gaps but located below this layer were excluded, and χ_*S*,*s*_ for each supraparticle was calculated and the mean determined with corresponding error bars.

Supraparticle size distributions were measured via laser diffraction using MasterSizer 3000/Hydro 3000S (Malvern Panalytical) over 10 measurements for each sample.

### 5.7 Characterization of Supraparticle Cross‐Sections

To facilitate visual interpretation of particle distributions in fractured supraparticles, a color‐coded segmentation mask was applied to a representative SEM cross‐sectional image using the Trainable Weka Segmentation plugin in Fiji (ImageJ). The model was trained on manually annotated regions to distinguish small particles, large particles, fragments, and background. Pre‐processing steps such as contrast enhancement and Gaussian filtering were used to improve segmentation accuracy. The resulting classification was color‐coded with small particles shown in blue and large particles in red, as presented in Figure 3b. This segmentation was used exclusively for visualization purposes. For quantitative evaluation, the cross‐sectional image was independently divided into 11 concentric radial shells. Within each shell, only particles within the plane of the cross‐section were manually counted and assigned to the small or large particle species based on their projected size. Error bars represent the standard deviation of the mean of the number fractions of three individual supraparticles for each segment.

### 5.8 Fluorescence Profile Characterization of Fluorescent SiO_2_:SiO_2_ Supraparticles

The internal structure and primary particle distribution of the fluorescent supraparticles were analyzed by confocal laser scanning microscopy (TCS SP5, Leica Mikrosysteme Vertrieb GmbH). The supraparticles used in this study were spray‐dried assemblies containing fluorescent silica particles as the smaller particle population and larger, non‐fluorescent silica particles as the large particle population. For imaging, the supraparticles were first dispersed in deionized water and drop‐casted onto a standard optical glass cover slip and dried. A droplet of Zeiss Immersol W 2010 was placed directly onto the objective lens to improve imaging quality. The sample was imaged using the argon laser at 10% laser intensity. Excitation was performed using the 476 nm laser line at 15% intensity, chosen based on the proximity to the maximum excitation wavelength of FITC at 495 nm. The emission range was set to 500–550 nm, in accordance with the emission maximum of FITC at 519 nm. To reduce background noise and improve image quality, the detector gain was set to 1042 and the offset to –5%. Z‐stack images were acquired with a step size of 0.13 µm. To enhance signal‐to‐noise ratio in single focal plane images, 4× line averaging and 1× accumulation, as well as 1× frame averaging and 16× accumulation, were performed. For quantitative analysis of the fluorescence distribution, a customized Python script was used to evaluate the radial fluorescence intensity profile within individual supraparticles. The script determined the area fraction of the fluorescence signal along the particle curvature, allowing spatially resolved recording of fluorescence intensity.

### 5.9 Thermal Characterization of Bulk Number Fraction χ_*S*,*b* − *exp*_

Thermogravimetric analysis was conducted using a TGA 2 instrument (Mettler Toledo, USA) equipped with an integrated balance and a small furnace. Approximately 30 mg of sample material was placed in aluminum crucibles with a filling volume of 70 µL. The measurement protocol consisted of two consecutive heating programs. The initial heating program was used to dry the particles. For this purpose, the temperature was increased dynamically from 35 °C to 50 °C at a heating rate of 50 K min^−1^ and then held isothermally at 50 °C for 1 h. Afterwards, the sample was cooled to 35 °C. Subsequently, a second heating program was then started for PS decomposition. The temperature was ramped from 35 °C to 200 °C at 5 K min^−1^ and then further increased to 600 °C at 2 K min^−1^. The final temperature of 600 °C was held isothermally for 20 min. All measurements were performed under a constant nitrogen flow of 50 mL min^−1^.

### 5.10 Simulation Model

To simulate a single suspension droplet during spray drying, an unresolved CFD‐DEM approach was employed to model fluid–particle and particle–particle interactions. Specifically, Hertz–Mindlin contact forces [70], constant attractive and repulsive forces according to DLVO theory [71], the Di Felice drag force model [72], and a vertical capillary force [55] were applied. Due to limitations of the unresolved CFD‐DEM method, hydrodynamic interactions can only be captured in the form of drag force models, taking into account local particle concentrations. To model two fluid phases and liquid evaporation, the VOF method was used, depicting a spherical droplet throughout the entire drying process [55, 73]. Further details of the simulation model can be found in Section S1, and in previous work [46, 74].

## Author Contributions

**Nicolas Vogel**: resources, supervision, formal analysis, project administration, writing – review and editing, writing – original draft, validation, funding acquisition, conceptualization. **Silas Wolf**: investigation, writing – original draft, methodology, validation, visualization, writing – review and editing, formal analysis, data curation, supervision. **Carsten Schilde**: funding acquisition, writing – review and editing, validation, project administration, supervision, resources, software, conceptualization. **Frederic Rudlof**: investigation, writing – original draft, validation, visualization, writing – review and editing, formal analysis, data curation, supervision. **Jonathan Martín González**: investigation, visualization, formal analysis. **Sonja Schaller**: data curation, investigation, visualization.

## Conflicts of Interest

The authors declare no conflicts of interest.

## Supporting information




**Supporting File 1**: advs76284‐sup‐0001‐SuppMat.pdf.

**Supporting File 2**: advs76284‐sup‐0002‐VideoS1.mp4.

## Data Availability

The data that support the findings of this study are presented in the Manuscript and the Supporting Information. All used data is provided at Zenodo under the following DOI: https://doi.org/10.5281/zenodo.20340680.
