# Supplementary material for: Optimizing Stratification in Binary Colloidal Supraparticles
Source: Adv Sci (Weinh). 2026 Jun 28;13(52):e76284. doi: 10.1002/advs.76284 (PMC13336991; doi:10.1002/advs.76284)
Supplement: Supplementary file 1 [file ADVS-13-e76284-s001.pdf]

Supporting Information

Optimising Stratification in Binary Colloidal Supraparticles

Frederic Rudlof[1], Silas Wolf[2], Sonja Schaller[1], Jonathan Martín González[1], Carsten Schilde[2*] and Nicolas Vogel[1*]

[1]*Institute of Interfaces and Particle Technology, Friedrich-Alexander-Universität Erlangen-Nürnberg, Erlangen, Germany*
[2]*Institute for Particle Technology, Technische Universität Braunschweig, Braunschweig, Germany*
[*]*Corresponding authors: c.schilde@tu-braunschweig.de, nicolas.vogel@fau.de*

Section S1: To consider two fluid phases, the volume of fluid method was applied. For unresolved VOF-DEM coupling (unresolved meaning CFD cells are significantly larger than the individual particles), the void fraction ε (fluid volume fraction) is used to account for the particles in each CFD cell, while α describes the volume fraction of liquid in each CFD cell ($\alpha = 0$ means only gas, $\alpha = 1$ means only liquid, $0<\alpha<1$ describes interface cells). ε is used in the continuity and momentum equations:

$$\frac{\partial \varepsilon_f}{\partial t} + \nabla \cdot (\varepsilon_f u_f) = 0 \tag{1}$$

$$\frac{\partial \varepsilon_f \rho_f u_f}{\partial t} + \nabla \cdot (\varepsilon_f \rho_f u_f u_f) = -\varepsilon_f \nabla p_{rgh} + \varepsilon_f \nabla \cdot \tau - \varepsilon_f g \cdot x \nabla \rho_f + \sigma \kappa \nabla \alpha \tag{2}$$

The position of the interface can be tracked with the α transport equation

$$\frac{\partial \alpha \varepsilon_f}{\partial t} + \nabla \cdot (\alpha \varepsilon_f u_f) - \nabla \cdot (u_c \alpha (1-\alpha)) = \dot{\alpha} \varepsilon_f \tag{3}$$

where $\dot{\alpha}$ describes the evaporation term which is calculated in the following way using a prescribed drying rate ψ, the cell length l_c, which is calculated using the volume of one cubic cell $l_c = \sqrt[3]{V_c}$, and a correction factor to account for the smearing of the interface $f_c = 2 \nabla\alpha\, l_c$:

$$\dot{\alpha} = -\frac{\psi \kappa}{16 \sqrt[3]{V_c}} f_c \tag{4}$$

$\dot{\alpha}$ is scaled by a factor f_c of 1500 to keep the simulation in computationally feasible limits and avoid numerical difficulties in form of parasitic currents.

The motion of the individual particles (velocity v_p and angular velocity ω_p of the particles dependent on their mass m_p and moment of inertia I_p) is calculated via DEM by estimating the acting forces and torques on the individual particles:

$$m_p \frac{dv_p}{dt} = F_{cont} + F_{DLVO} + F_{drag} + F_{cap} \tag{5}$$

$$I_p \frac{d\omega_p}{dt} = M_{pp} \tag{6}$$

The forces taken into account can be grouped into particle-particle forces, namely the contact force F_{cont} based on the Hertz-Mindlin model[1] and the DLVO model F_{DLVO} that describes the combination of attractive van der Waals force F_{VdW} and repulsive interaction force F_{el} due to the electrostatic double layer[2]:

$$F_{cont} = -\frac{4}{3} E^* \sqrt{R^*}\, \delta_n^{3/2} \tag{7}$$

$$F_{DLVO} = F_{VdW} + F_{el} = \left(-\frac{\overline{R^*}}{2} A \frac{1}{6x^2} + \frac{R^*}{2} \lambda K e^{-\lambda x} \right) \overline{x} \tag{8}$$

where E^* and R^* are used for the equivalent Young's modulus and radius of the interacting particles, δ_n is their normal overlap, A is the Hamaker constant, x is the distance of the two particles and λ is the inverse Debye length. K is the interaction constant dependent, among others, on the zeta potential ζ. $\overline{x}$ represents the distance unit vector. Given that purified water was used for the dispersions and no additional salts were introduced, an ionic strength of 5 mM was assumed to account for trace ions, the autodissociation of water, and dissolved CO_2. This yields a Debye length of approximately 4 nm, which was held constant throughout the drying process. This rough estimate is considered reasonable, as the energy barrier is governed primarily by the high absolute zeta potentials (-54 mV for SiO_2; -45 mV for PS), and neither the absolute value of the ionic strength nor its changes during drying are expected to significantly alter the particle interaction regime, given the limited droplet shrinkage during the first drying stage as a result of the high solids concentration. Additionally, particle-fluid forces are employed such as the drag force F_{drag} according to the model by Di Felice[3] and a capillary force F_{cap} that pushes the particles away from the fluid-fluid interface using only the radial component and omitting the lateral component[4]:

$$F_{drag} = 0.125\, C_D\, \rho_f\, \pi\, d_p^2\, \varepsilon_f^2 \left| u_f - v_p \right| \left(u_f - v_p \right) \varepsilon_f^{-\chi} \tag{9}$$

$$F_{cap} = 2\pi\, r_P\, \sigma \sin(\omega) \sin(\omega + \theta)\, n \tag{10}$$

using the drag coefficient C_D and an exponential factor χ dependent on the Reynolds number. The capillary force depends on the particle radius r_P, the wetting angle ω, the contact angle θ and acts in direction of the interface unit normal vector n. Both particle-fluid forces were treated using a one-way coupling approach, meaning that the forces exerted on the particles were not reflected back onto the fluid. As discussed in an earlier publication,[5] Brownian motion was not taken into account due to the high Péclet number in spray drying applications making diffusive effects irrelevant compared to advective motion.

Particles were initialised in a spherical domain with theoretically determined sizes according to the experiments and the given concentration and volume fractions. A sphere of 30 µm in diameter was defined in the CFD and assigned with the respective α values to represent the droplet. The spherical shape was maintained throughout the simulation, corroborating the consolidated, spherical supraparticles observed experimentally.[6] The mesh resolution was chosen depending on the diameter of the largest particle (cell length $l_c = 1.5\, d_p$) to ensure the resolution to be as high as possible while obeying the unresolved CFD-DEM condition ($l_c > d_p$). The mesh dimensions in each direction had a length of 1.5 d_d dependent on the droplet diameter d_d ensuring enough space between the droplet boundaries and the domain boundaries to avoid numerical artefacts. Further details on the models and simulation procedure are available in our previous publications.[5,7]

Section S2: To derive $\chi_{S,b-exp}$, TGA measurements were performed. The relevant weight loss range of 250 °C – 600 °C was derived by differential thermogravimetry (DTG). From TGA curves of pure PS and SiO_2 supraparticles, the weight loss of PS corresponding to the smaller particle species was determined to be 100% and for the weight loss of SiO_2, ω_{Sio2}, being the larger particle, 3.00524% in the relevant weight loss range. The obtained PS weight loss, ω_{PS}, in the respective range for the binary SP was defined as:

$$\omega_{PS} = \omega_{total} - 3.00524 \cdot \frac{\phi_{Sio2} \cdot \rho_{Sio2}}{(\phi_{PS} \cdot \rho_{PS} + \phi_{Sio2} \cdot \rho_{Sio2})} \tag{1}$$

Here, ω_{total} is the observed weight loss measured in the TGA analysis.

To retrieve the volume fraction of PS, ϕ_{PS}, in the experimental system, we must consider the density of PS ρ_{PS} and SiO_2 ρ_{Sio2}. ϕ_{PS} is defined as:

$$\phi_{PS} = \frac{\frac{\omega_{PS}}{\rho_{PS}}}{\frac{\omega_{PS}}{\rho_{PS}} + \frac{1-\omega_{PS}}{\rho_{Sio2}}} \tag{2}$$

In general, the volume ratios can be converted into number ratios of small and large particles, χ_S and χ_L, respectively, as follows:

$$\frac{\chi_S}{\chi_L} = \frac{\phi_S}{\phi_L} \cdot \left(\frac{d_L}{d_S}\right)^3 \tag{3}$$

χ_S can then be derived as:

$$\chi_S = \frac{\phi_S \left(\frac{d_L}{d_S}\right)^3}{1 - \phi_S + \phi_S \left(\frac{d_L}{d_S}\right)^3} \tag{4}$$

χ_S is equivalent to $\chi_{S,b}$ as it describes the number fraction of particles within the entire supraparticle. The equation in this form corresponds to the generic form of Eq. 1 in the main text. The equation can be utilised to calculate the theoretical number fraction $\chi_{S,b}$ or the experimental number fraction derived from TGA measurements, $\chi_{S,b-exp}$ by inserting the theoretical value for ϕ_S, or the experimentally determined ϕ_{PS}.

To parameterise the observed number-based enrichment of small colloids as a continuous function of particle size ratio, we employed an empirical fitting function by extending the retrieved model with additional offset and scaling parameters to get:

$$\chi_S = \frac{A * \phi_S * \left(\left(\frac{d_L}{d_S}\right) - x_0\right)^3}{1 - \phi_S + A * \phi_S \left(\left(\frac{d_L}{d_S}\right) - x_0\right)^3} + b \tag{5}$$

With A for scaling of the amplitude, ϕ_S as the effective volume fraction, x_0 as correction term for x-shift and b as offset in y-direction. Minor deviations between experimental and theoretical trends are observed, which may arise from inaccuracies in volume fraction control, polydispersity effects, or local transport fluctuations during droplet drying. The added degrees of freedom in the function allowed for

more accurate representation of the experimental data without imposing strict symmetry or saturation assumptions.

Section S3: The predictive tool for surface enrichment is available as an interactive tool to support the targeted design of supraparticles. It can be directly accessed via the following link:

https://mybinder.org/v2/gh/SilasWolf/interactive-segregation-tool.git/main?urlpath=voila%2Frender%2Finteractive_tool.ipynb

Furthermore, the Python code as well as Jupyter Notebook file can be accessed via the following GitHub repository or as .zip file as supporting material to this publication:

https://github.com/SilasWolf/interactive-segregation-tool.git

Table S1: Péclet numbers of d_S (PS) and d_L (SiO_2) according to their particle diameter and size ratio d_L/d_S. The Péclet numbers were calculated after Vehring et al.[8] as: $\mathrm{Pe}_i = \psi_d/8D_i$. The drying rate was estimated based on results from Vehring et al.[8] according to the spray drying parameters in this study.

d_S (PS) / nm	d_L (SiO_2) / nm	d_L/d_S / -	Pe (PS) / -	Pe (SiO_2) / -
396	390	1.0	1293	1273
611	607	1.0	1994	1981
396	436	1.1	1293	1423
611	676	1.1	1994	2207
402	489	1.2	1312	1596
315	476	1.5	1028	1554
485	751	1.5	1583	2451
270	500	1.9	881	1632
303	620	2.0	989	2024
303	1000	3.3	989	3264
270	900	3.3	881	2938
268	1091	4.0	875	3561
129	649	5.0	421	2118
134	843	6.3	437	2752
129	900	7.0	421	2938
129	958	7.4	421	3127

Table S2: Volume-weighted median diameter of the formed supraparticles for all 16 formulations (rows: ϕ_S = 1, 5, 15, 30%; columns: d_L/d_S= 1, 3, 5, 7). d_L/d_S were derived from the mean diameter of PS (used as the small particles, with a diameter d_S) and SiO_2 (used as the large particles, with a diameter d_L).

d_S	d_L	d_L/d_S	***d₅₀*(*ϕ*S = 1%)**	***d₅₀*(*ϕ*S = 5%)**	***d₅₀*(*ϕ*S = 15%)**	***d₅₀*(*ϕ*S = 30%)**
[nm]	[nm]	-	[µm]	[µm]	[µm]	[µm]
396	390	0.98		15.03	16.19	20.94
611	607	0.99	18.57	-	-	-
270	900	3.33	-	18.14	14.46	17.71
303	1000	3.30	17.25	-	-	-
129	649	5.03	17.05	18.52	19.76	26.23
129	900	6.98	-	11.93	16.32	14.12
129	958	7.43	13.94	-	-	-

Table S3: Theoretical and measured TGA results and binary compositions of SiO_2:PS supraparticles. ω_{PS} for ϕ_S = 1%, 5%, 15%, and 30% for d_L/d_S = 1–7. Respective theoretical $\chi_{S,b}$ and experimental $\chi_{S,b-exp}$ are derived by ω_{PS}.

ϕ_S	ϕ_L	d_L/d_S	ω_{PS} (theor)	ω_{PS} (exp)	ω_{SiO2} (theor)	$\omega_{PS+SiO2}$ (theor)	$\omega_{PS+SiO2}$ (exp)	$\chi_{S,b}$	$\chi_{S,b-exp}$	χ_S/χ_L (exp)
%	%	-	%	%	%	%	%	%	%	-
1	99	0.99	0.53	0.80	2.99	3.52	3.79	0.98	1.49	0.015
		1.10		0.77			3.76	1.35	1.96	0.020
		1.21		0.64			3.63	1.79	2.16	0.022
		1.55		0.41			3.40	3.61	2.81	0.028
		2.05		0.69			3.68	7.96	10.18	0.11
		3.30		0.55			3.54	26.64	27.49	0.38
		4.07		0.36			3.35	40.53	31.51	0.46
		5.03		0.46			3.45	56.26	52.80	1.12
		6.29		0.34			3.33	71.55	61.68	1.61
		7.43		0.72			3.71	80.54	85.06	5.70
5	95	0.98	2.69	3.22	2.92	5.61	6.15	4.79	5.71	0.061
		1.10		4.42			7.34	6.56	10.51	0.12
		1.22		3.43			6.36	8.65	10.86	0.12
		1.51		3.14			6.06	15.37	17.56	0.21
		1.85		2.05			4.97	25.05	20.19	0.25
		3.33		2.39			5.32	66.09	63.37	1.73
		4.07		2.57			5.49	78.03	77.19	3.38
		5.03		2.57			5.50	87.02	86.52	6.42
		6.29		2.48			5.41	92.91	92.35	12.08
		6.98		3.05			5.98	94.70	95.32	20.36
15	85	0.98	8.48	8.70	2.75	11.22	11.15	14.43	14.77	0.17
		1.10		11.25			13.99	19.06	24.37	0.32
		1.22		9.00			11.75	24.11	25.33	0.34
		1.51		8.61			10.95	37.85	38.22	0.62
		1.85		6.88			9.62	52.85	47.20	0.89
		3.33		7.60			10.38	86.73	85.29	5.80
		4.07		8.15			10.88	92.25	91.93	11.39
		5.03		8.76			11.50	95.74	95.88	23.30
		6.29		8.13			10.88	97.77	97.67	42.00
		6.98		8.81			10.57	98.36	98.43	62.52
30	70	0.98	18.37	18.20	2.45	20.82	20.24	29.05	28.82	0.41
		1.10		22.74			24.94	36.39	42.80	0.75
		1.22		18.59			21.04	43.55	43.92	0.78
		1.51		17.88			19.38	59.66	58.87	1.43
		1.85		15.19			17.63	73.13	68.42	2.17
		3.33		17.35			19.59	94.07	93.68	14.81
		4.07		17.75			20.19	96.66	96.52	27.72
		5.03		19.08			21.53	98.20	98.28	57.20
		6.29		17.69			20.13	99.07	99.03	101.9
		6.98		18.51			20.96	99.32	99.32	147.0

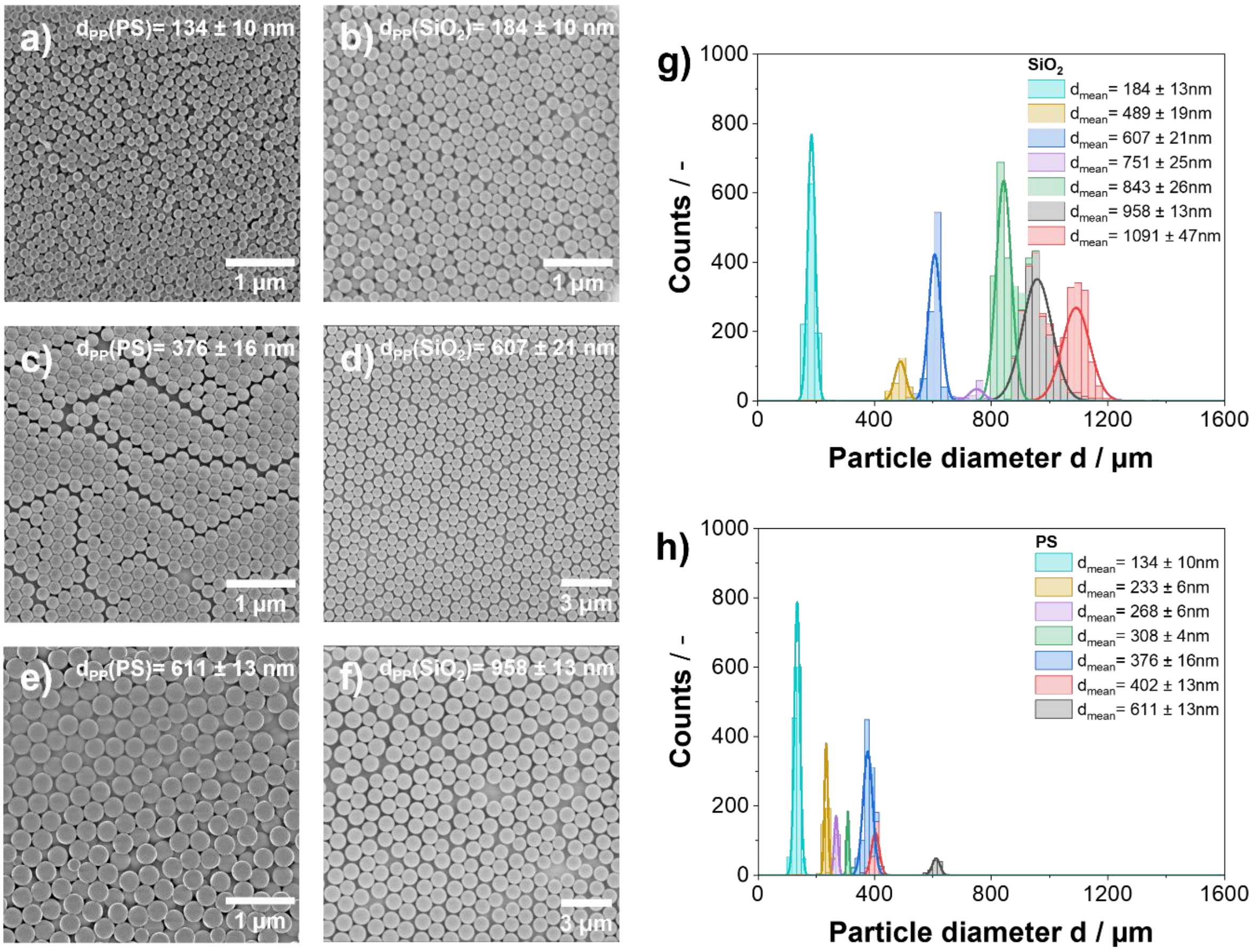

Figure S1: Colloidal SiO_2 and PS primary particles. a,c,e) SEM images of PS primary particles with d_{mean} of a) 134 nm, c) 376 nm and e) 611 nm. b, d, f) SEM images of SiO_2 primary particles with d_{mean} of b) 184 nm, d) 607 nm and f) 958 nm. g,h) Corresponding SEM particle size distribution of g) PS particles and h) SiO_2 particles.

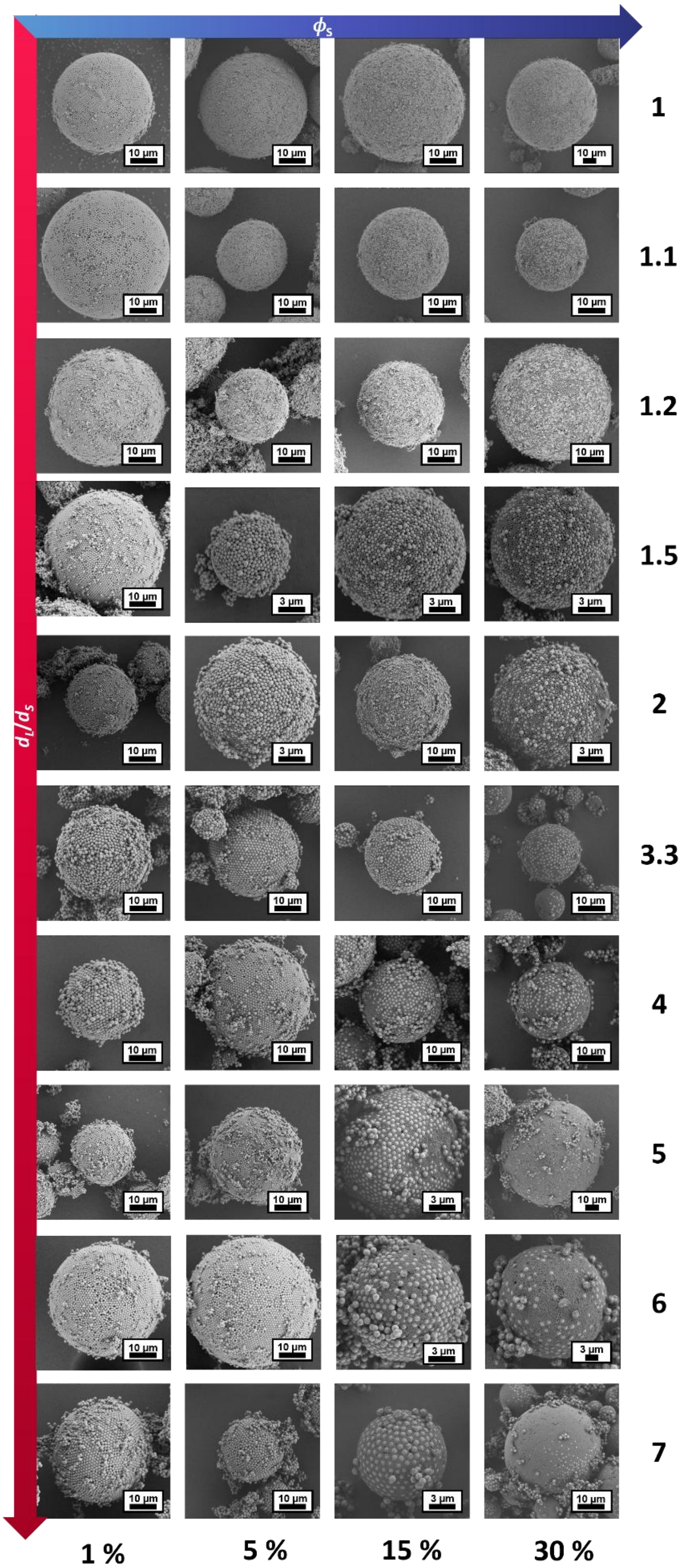

Figure S2: Top-view SEM images of binary SiO_2:PS supraparticles for corresponding size ratios d_L/d_S and volume fractions of small particles ϕ_S. From left to right: Increasing size ratio d_L/d_S from 1–7. From top to bottom: Increasing volume fraction ϕ_S from 1–30%.

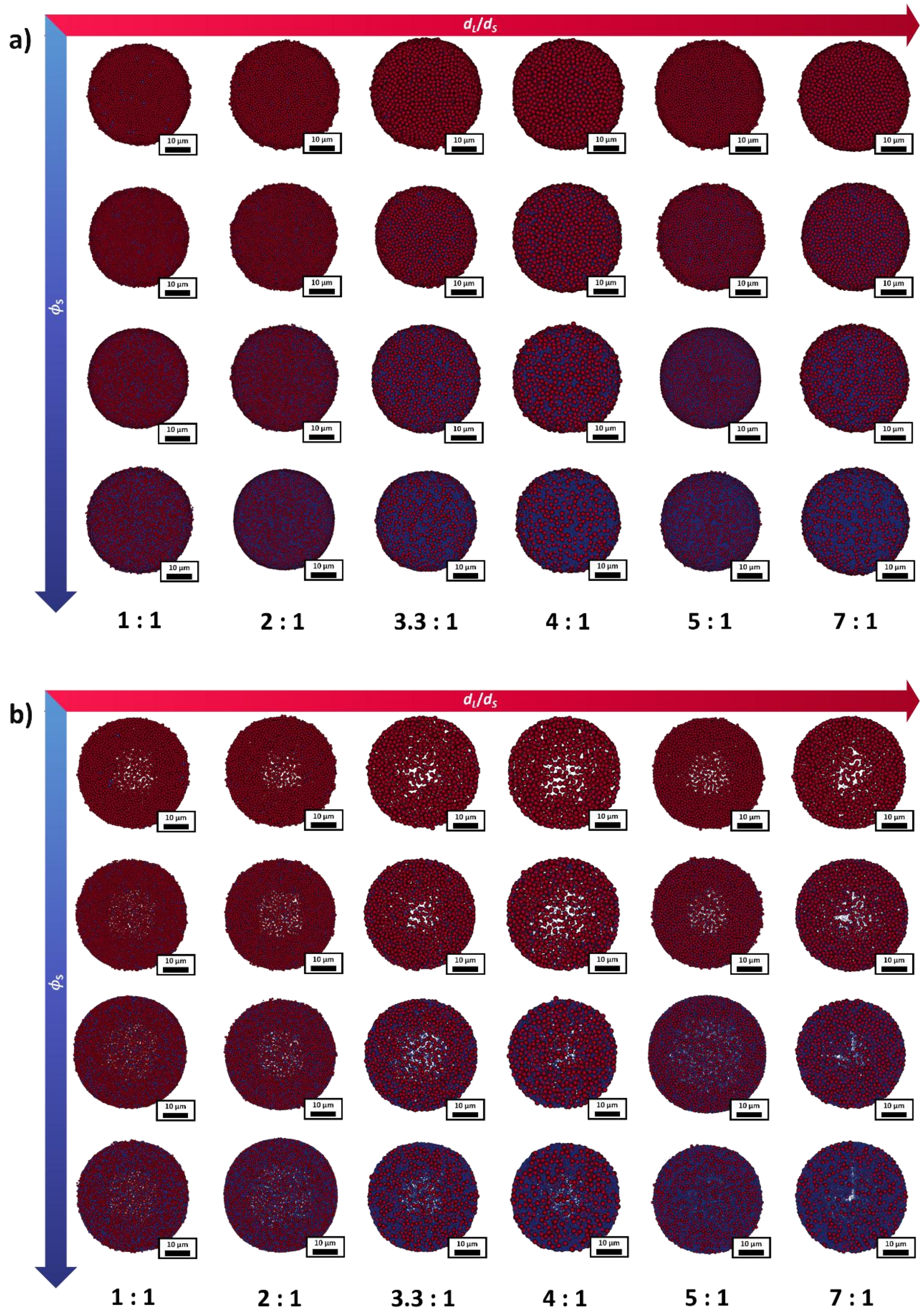

Figure S3: a) Top-view and b) cross-section of simulated binary supraparticles for corresponding size ratios d_L/d_S and volume fractions of small particles ϕ_S. Red particles indicate d_L, blue particles indicate d_S. From left to right: Increasing size ratio d_L/d_S from 1–7. From top to bottom: Increasing volume fraction ϕ_S from 1% to 30%.

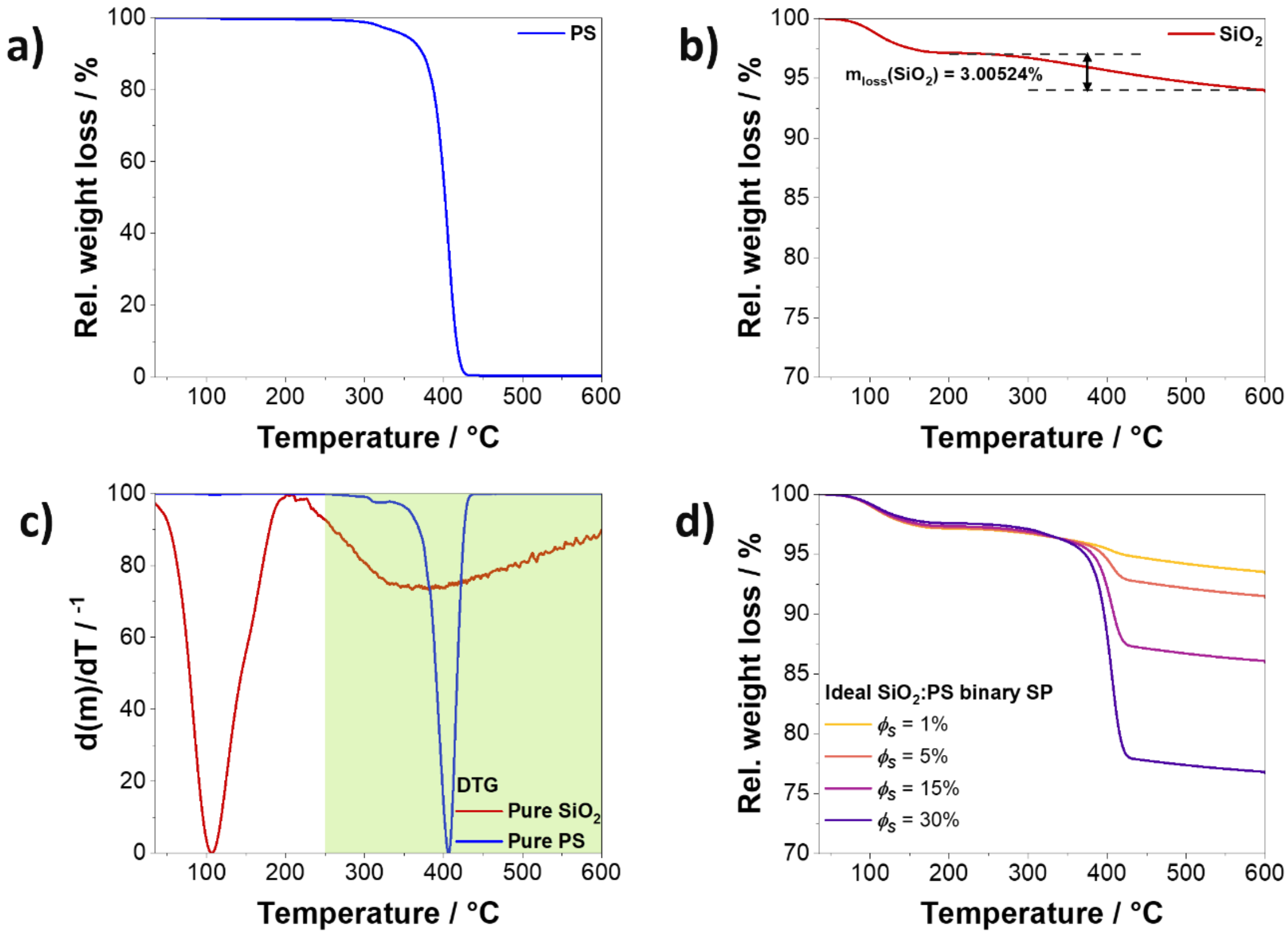

Figure S4: a,b) TGA measurements of PS SPs (a) and SiO_2 supraparticles (b). c) DTG curve of PS and SiO_2 supraparticles. d) Theoretical curves of binary SiO_2:PS supraparticles with ϕ_S from 1% to 30% derived from a) and b).

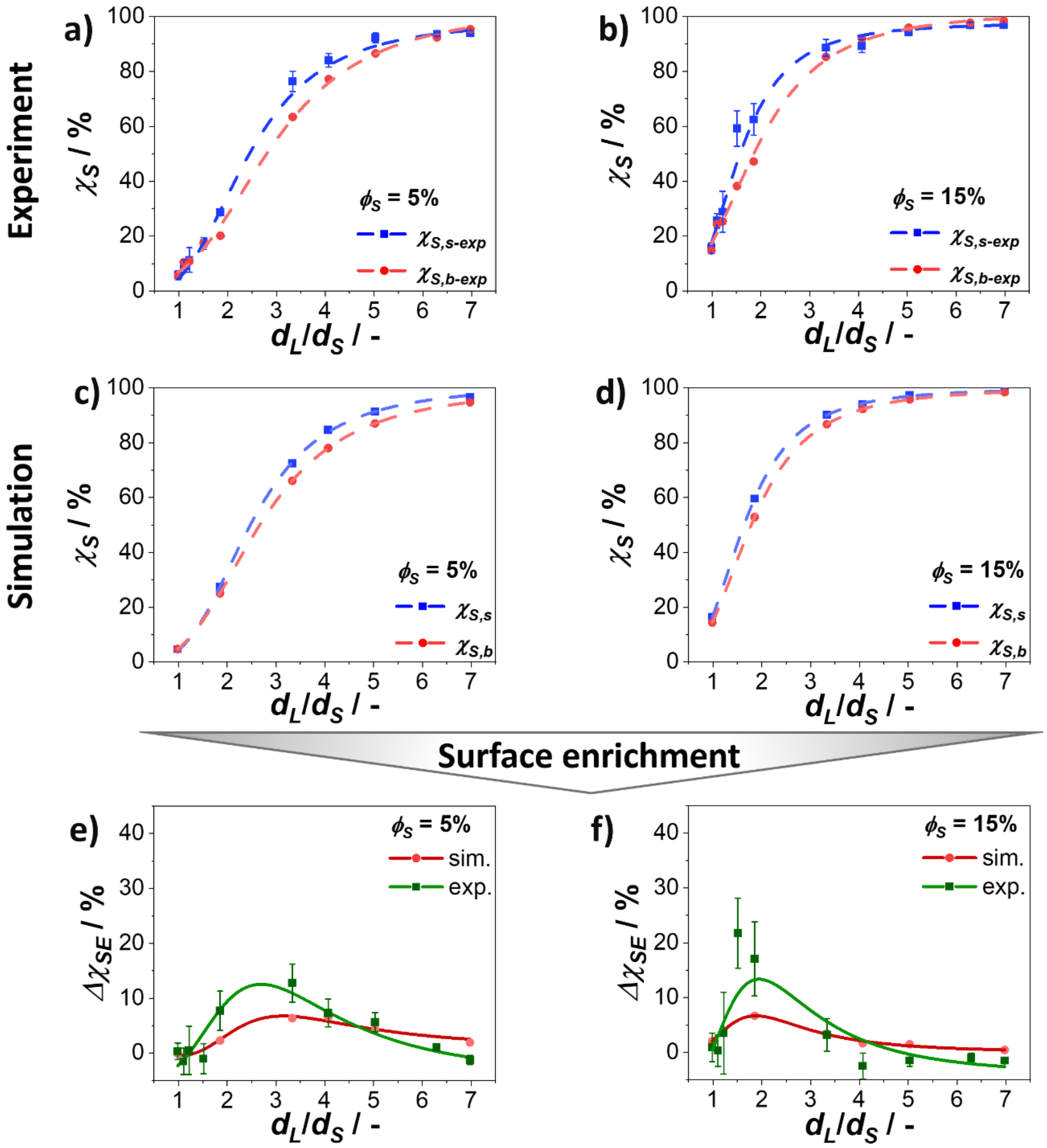

Figure S5: Dependence of $\Delta\chi_{SE}$ on d_L/d_S for $\phi_S = 5\%$ and $\phi_S = 15\%$. a,b) Experimentally derived $\chi_{S,b-exp}$ and $\chi_{S,s-exp}$ from image analysis of supraparticle surface for (a) $\phi_S = 5\%$ and (b) $\phi_S = 15\%$. c,d) Corresponding simulations for (c) $\phi_S = 5\%$ and (d) $\phi_S = 15\%$. e,f) Surface enrichment $\Delta\chi_{SE}$ for e) $\phi_S = 5\%$ and f) $\phi_S = 15\%$. Green data points are experimental data for $\Delta\chi_{SE}$, red data points show simulation data for $\Delta\chi_{SE}$. The respective lines are the differences between the fit functions of $\chi_{S,s}$ and $\chi_{S,b}$ from simulations and experiment.

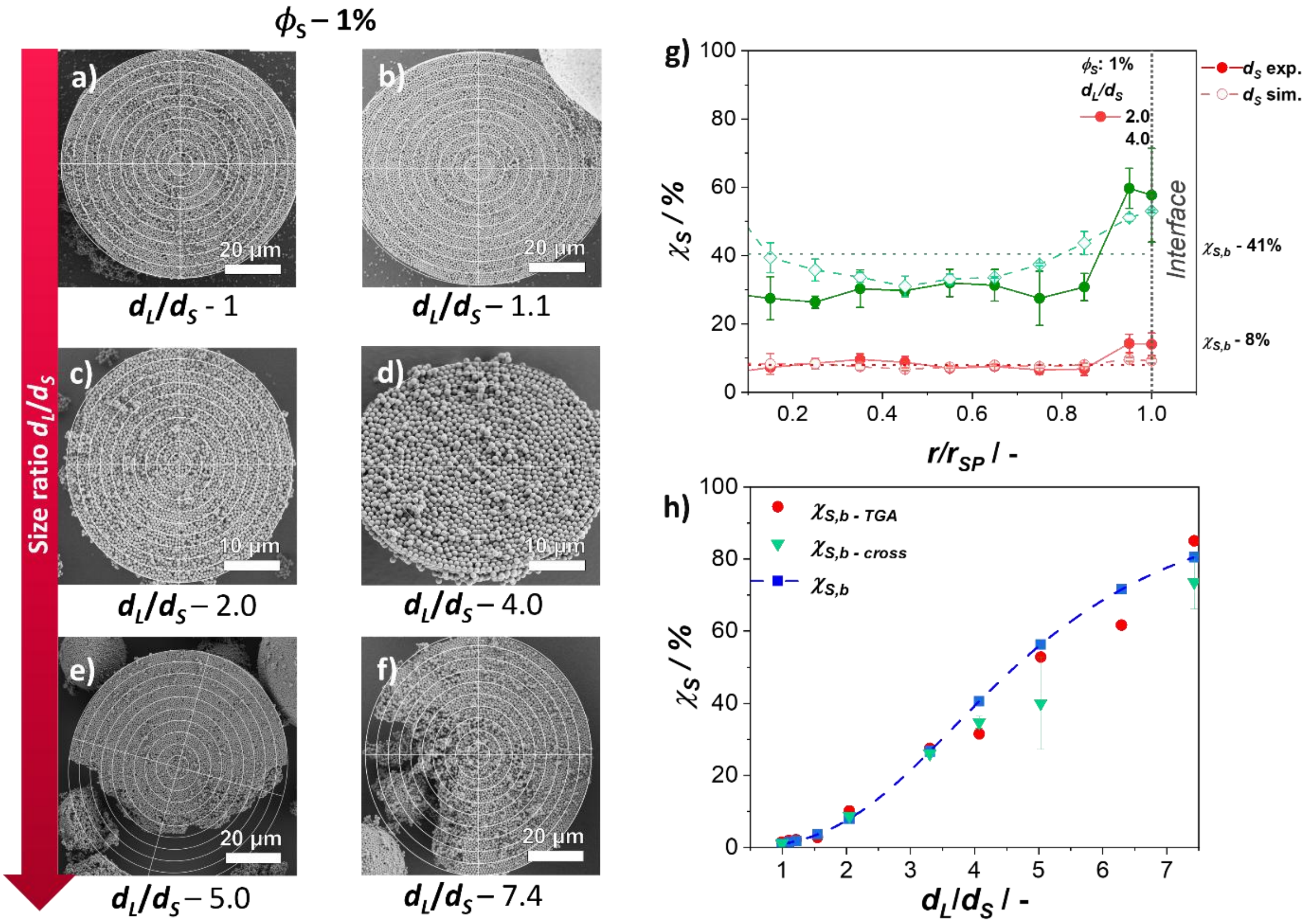

Figure S6: a-f) Cross-sectional SEM images of supraparticles formed at ϕ_S = 1% and d_L/d_S of: a) 1, b) 1.1, c) 2.0, d) 4.0, e) 5.0 and f) 7.4. g) Segmentational characterisation of the number fraction of small particles, χ_S. h) Number fraction $\chi_{S,b-exp}$ (red), $\chi_{S,b-cross}$ from cross-sectional image analysis (green) and $\chi_{S,b}$ for $\phi_S = 1\%$. Note that $\chi_{S,b-cross}$ underestimates $\chi_{S,b}$ with increasing d_L/d_S (> 3.3) caused by the evaluation method, in which only the topmost visible small particles within the continuously densely packed cross-sectional plane are considered.

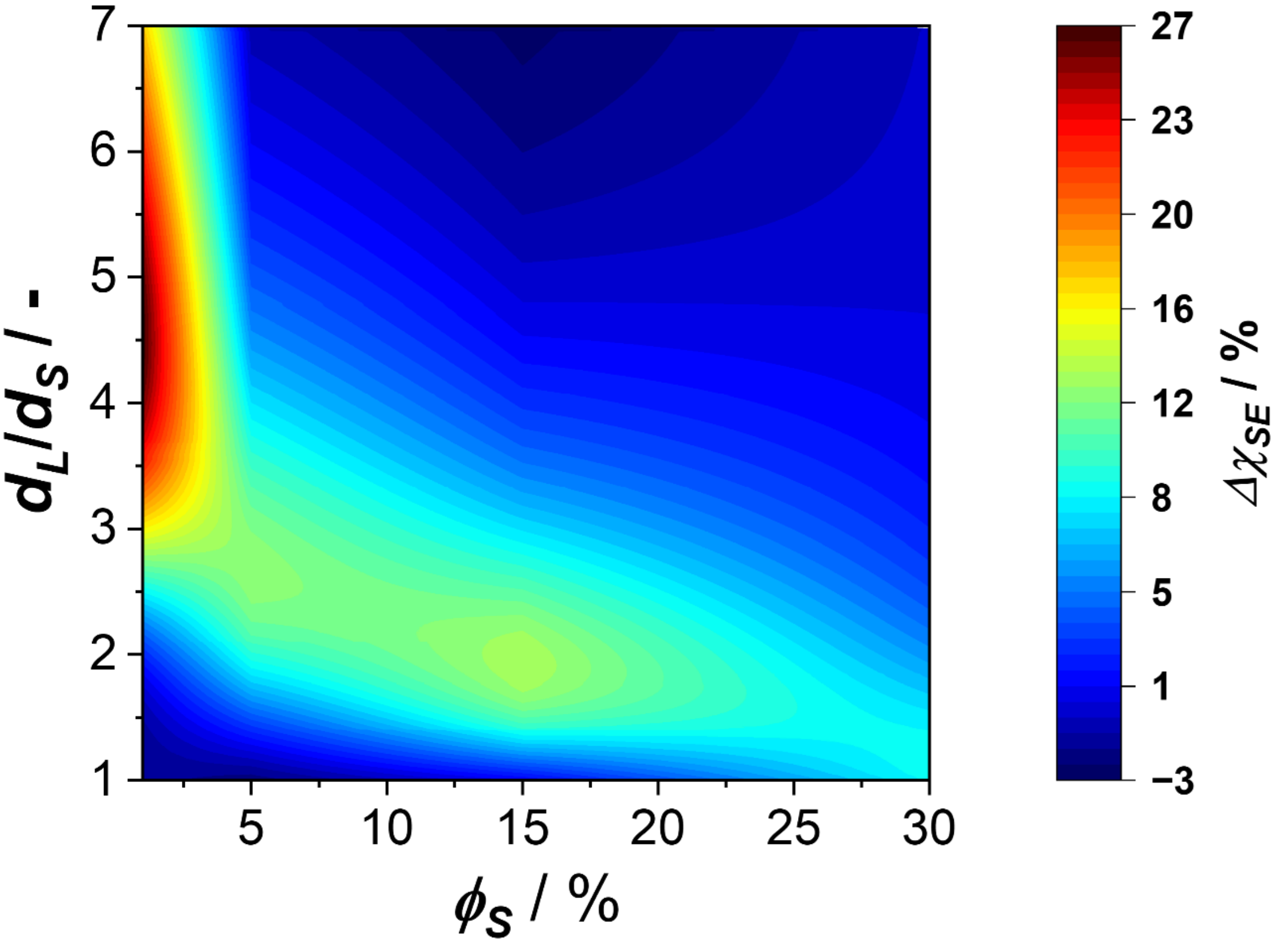

Figure S7: Experimentally derived contour plot of $\Delta\chi_{SE}$ as a function of ϕ_S and d_L/d_S.

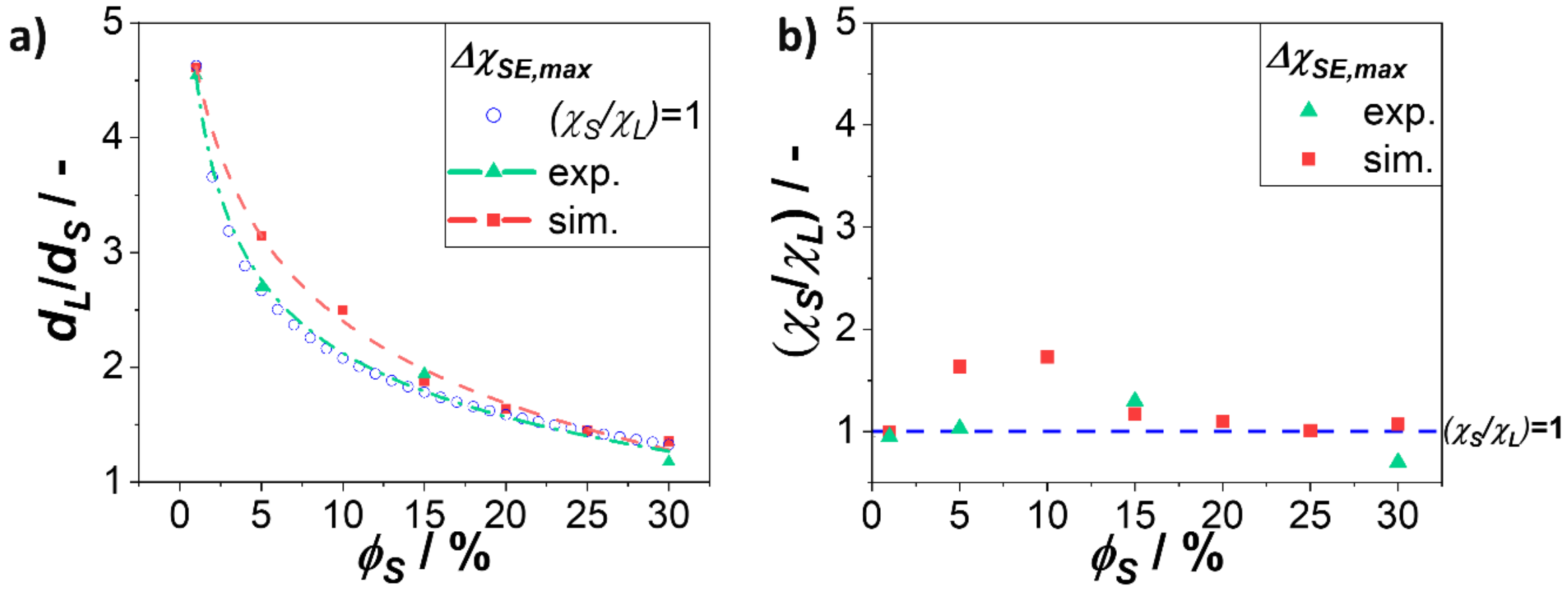

Figure S8: a) $\Delta\chi_{SE,max}$ for d_L/d_S at different relative volume fraction of small particles, $\phi_S = 1 - 30\%$ derived from simulation (red), experiments (green) and theoretically derived $\Delta\chi_{SE}$ after $d_L/d_S = (\chi_S/\chi_L * (1-\phi_S)/\phi_S)^{1/3}$ for $\chi_S/\chi_L \approx 1$ (blue). b) χ_S/χ_L at $\Delta\chi_{SE,max}$ for $\phi_S = 1 - 30\%$ for simulation (red), experiments (green). The dashed blue line indicates $\chi_S/\chi_L = 1$.

Supplementary Videos:

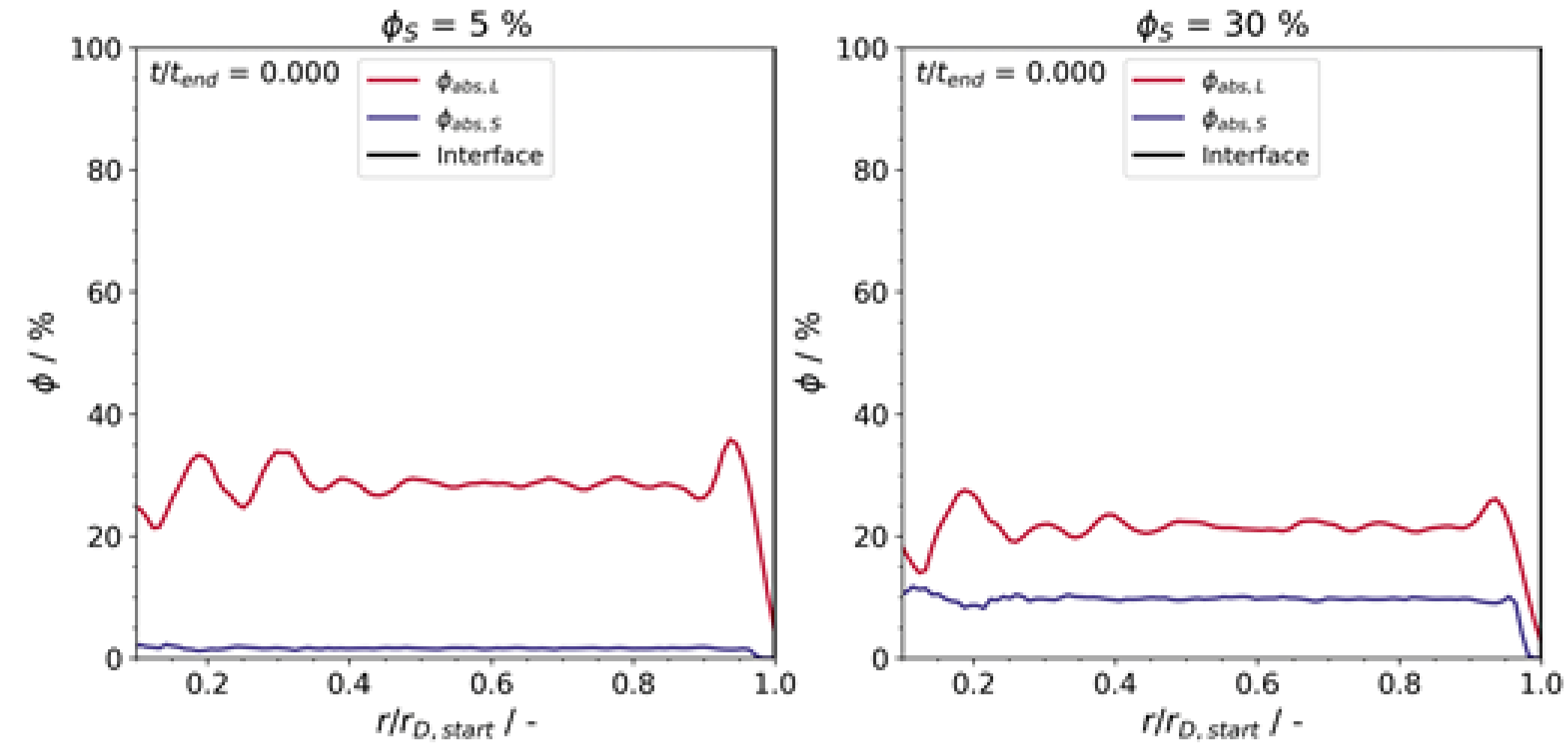

Supplementary Video 1: Temporal evolution of the local volume fractions $\phi_{abs,S}$ (small particles, blue line) and $\phi_{abs,L}$ (large particles, red line) along their radial position with respect to the initial droplet radius $r_{D,start}$ during supraparticle assembly. The black line indicates the position of the receding liquid-air interface. $t/t_{end} = 0$ corresponds to the initial dispersion volume content of 28 vol.%, $t/t_{end} = 1$ to the fully consolidated supraparticle.

1. A. Di Renzo, and F. P. Di Maio, "An Improved Integral Non-Linear Model for the Contact of Particles in Distinct Element Simulations," *Chemical Engineering Science* 60, no. 5 (2005): 1303. https://doi.org/10.1016/j.ces.2004.10.004.

2. J. N. Israelachvili 2011 *Intermolecular and Surface Forces.* Third Ed., Waltham, MA, USA: Academic Press. ISBN 9780123751829.

3. R. Di Felice, "The Voidage Function for Fluid–Particle Interaction Systems," *International Journal of Multiphase Flow* 20, no. 1 (1994): 153. https://doi.org/10.1016/0301-9322(94)90011-6.

4. T. Breinlinger, A. Hashibon, and T. Kraft, "Simulation of the Influence of Surface Tension on Granule Morphology during Spray Drying Using a Simple Capillary Force Model," *Powder Technology* 283 (2015): 1. https://doi.org/10.1016/j.powtec.2015.05.009.

5. S. Wolf, N. Kühn, D. Ivanov, A. Levy, and C. Schilde, "Numerical Study of Particle Segregation during Spray Drying of Binary Suspension Droplets," *Powder Technology* 459 (2025): 121019. https://doi.org/10.1016/j.powtec.2025.121019.

6. R. Clift, J. R. Grace, M. E. Weber 2013 *Bubbles, Drops, and Particles.* First Ed., Mineola, NY, USA: Dover Publications. ISBN 9780486317748.

7. S. Wolf, M. Lippke, A. Schoo, A. Kwade, and C. Schilde, "A Computational Fluid Dynamics–Discrete Element Method Model for Physics-Based Simulation of Structure Formation during Battery Electrode Drying," *Energy Technology* 12, no. 4 (2024): 2301004. https://doi.org/10.1002/ente.202301004.

8. R. Vehring, W. R. Foss, and D. Lechuga-Ballesteros, "Particle Formation in Spray Drying," *Journal of Aerosol Science* 38, no. 7 (2007): 728. https://doi.org/10.1016/j.jaerosci.2007.04.005.
